# Early-Stage Pancreatic Cancer Diagnosis: Serum Biomarkers and the Potential for Aptamer-Based Biosensors

**DOI:** 10.3390/molecules30092012

**Published:** 2025-04-30

**Authors:** Weisi He, Jingyu Cui, Xue-Yan Wang, Ryan H. P. Siu, Julian A. Tanner

**Affiliations:** 1 School of Biomedical Sciences, LKS Faculty of Medicine, The University of Hong Kong, Hong Kong SAR, China; weisi.he.31@connect.hku.hk (W.H.); cjy1996@connect.hku.hk (J.C.); wangxyyy@connect.hku.hk (X.-Y.W.); u3008168@connect.hku.hk (R.H.P.S.); 2 Advanced Biomedical Instrumentation Centre, Hong Kong Science Park, Hong Kong SAR, China; 3 Materials Innovation Institute for Life Sciences and Energy (MILES), HKU-Shenzhen Institute of Research and Innovation (HKU-SIRI), Shenzhen 518057, China

**Keywords:** early-stage pancreatic cancer diagnosis, biomarker, aptamer, sensor

## Abstract

Pancreatic cancer has a high mortality rate, and both the incidence and mortality are continuing to increase in many countries globally. The poor prognosis of pancreatic cancer is in part due to the challenges in early diagnosis. Improving early-stage pancreatic cancer diagnosis would improve survival outcomes. Aptamer-based biosensors provide an alternative technological approach for the analysis of serum biomarkers with several potential advantages. This review summarizes the major pancreatic cancer serum biomarkers, as well as discusses recent progress in biomarker exploration and aptasensor development. Here, we review both established and novel serum biomarkers identified recently, emphasizing their potential for early-stage pancreatic cancer diagnosis. We also propose strategies for further expanding multiplex biomarker panels beyond the established CA19-9 biomarker to enhance diagnostic performance. We discuss technological advancements in aptamer-based sensors for pancreatic cancer-related biomarkers over the last decade. Optical and electrochemical sensors are highlighted as two primary modalities in aptasensor design, each offering unique advantages. Finally, we propose steps towards clinical application using aptamer-based sensors with multiplexed biomarker detection for improved pancreatic cancer diagnostics.

## 1. Introduction

Pancreatic cancer (PC), one of the six primary gastrointestinal cancers, has a high mortality rate with a poor prognosis. Incidence rates and death rates have increased globally since the year 2000 [1]. In China, the incidence rate has increased significantly over the last two decades and remains on an upward trajectory [2]. In 2020 and 2021, Europe and the Americas occupied the top two highest incidence and death rates for global PC [1]. In the United States, for 2025, it has been estimated that PC will be the third-ranking cancer ranked by total deaths and the least favorable in prognosis [3]. The 5-year survival rate is only 8% [3]. This poor prognosis of PC is partially due to the late detection of PC [4]. Overall, the 5-year survival rate at least doubles for PC patients after surgical resection [5]. Less than 20% of patients can confirm PC at a stage where surgical resection can be performed [5]. Improving early-stage detection is one of the most effective strategies for reducing the PC mortality rate [4].

At present, tissue biopsy, imaging, and blood tests are three major diagnostic methods for PC. Tissue biopsy is the most sensitive method among these three primary methods, but it is expensive, not routinely advised, and carries risks due to invasive surgery [6]. Imaging and blood tests are cheaper and almost non-invasive, but usually the sensitivity is insufficient [6]. Compared to imaging, blood testing carries convenience for patients, requires less equipment, and has the potential to distinguish the grade of pancreatic intraepithelial neoplasia (PanIN), the precursor of PC [5]. High-sensitivity and specificity serum biomarkers are needed for early PC detection.

To date, there are no blood-based diagnostic tools for early-stage pancreatic cancer that have received FDA approval for clinical use. Several multi-target blood-based tests have obtained Clinical Laboratory Improvement Amendments (CLIA) certification for market availability, including the GRAIL test [7], Avantect Pancreatic Cancer test [8], IMMray PanCan-d test [9], and Toray APOA2-iTQ test [10,11]. The GRAIL test employs cell-free DNA sequencing combined with machine learning, demonstrating relatively low sensitivity (14.5–44.1%) in early-stage PC but high sensitivity (73.4–92.2%) in advanced stages [7]. The Avantect Pancreatic Cancer test targets PC through next-generation sequencing of 5-hydroxymethylation signatures and machine learning, achieving moderate sensitivity (68.3%) in early-stage disease [8]. The IMMray PanCan-d test, the first commercially available blood-based PC assay (withdrawn in 2023), uses an antibody microarray platform to measure CA19-9 and eight biomarkers, yielding good sensitivity (85–89%) for early-stage PC [9]. The Toray APOA2-iTQ test applies enzyme-linked immunosorbent assays (ELISAs) to detect apolipoprotein A2 isoforms, with moderate sensitivity (50%) in early-stage PC, which improves to 70% when combined with CA19-9 testing [11]. While these assays generally claim high sensitivity for late-stage pancreatic cancer, their performance in early detection remains suboptimal. Additionally, these tests require costly equipment or consumables, limiting their accessibility and cost-effectiveness for screening populations.

In current clinical applications, blood-based assays widely use antibody techniques, such as an enzyme-linked immunosorbent assay (ELISA). Compared to antibodies, aptamers have advantages including superior stability that facilitates easier storage and extends shelf life, are generally less expensive, and, as they are chemically synthesized, have less batch-to-batch variation in manufacturing. Furthermore, aptamers possess a smaller molecular size and greater amenability to chemical modification, which is beneficial to detection sensitivity [12]. Aptamers are oligonucleotides with secondary structures that can interact with biomolecules, glycans, proteins, or exosomes. Aptamers can be easily chemically engineered to enhance their properties, such as stability, specificity, and binding affinity [12]. The small size and ease of modification lead aptamers to offer more potential for multiplexed simultaneous detection of multiple targets, which has the potential to increase detection sensitivity. Aptamer-based biosensors are a potential solution, combining the capabilities of sensitive aptamer molecular recognition with engineered stability and cost-effectiveness. Aptamers are an attractive strategy for clinical application or even for a point-of-care device in early PC diagnosis.

In the first section of this review, we summarize the serum biomarkers, including the most frequent traditional biomarkers and recently discovered biomarkers. The potential usage in early-stage PC diagnosis will be discussed. In the second section, we focus on the updated techniques for the development of aptamer-based sensors of PC biomarkers, especially focusing on techniques that could achieve clinical detection sensitivity or are adaptable for clinical environments.

## 2. Serum Biomarkers for Pancreatic Cancer

Biomarkers are specific biomolecules that change along with the disease status. Serum biomarkers for PC consist of proteins, glycans, extracellular vehicles (EVs), circulating cells (CTCs), circulating RNAs, circulating DNAs, lipids, and metabolites. Early-stage PC-related clinical serum biomarkers have value that needs to be further investigated in developing aptamers. For PC serum biomarkers, aptamers have been developed mainly against proteins due to the difficulty of targeting small molecules [13]. In this review, we do not consider complementary oligonucleotides that detect nucleic acids as aptamers and focus on nucleic acid aptamers that bind to non-nucleic acid targets. Hence, in the biomarker section, we will mainly focus on introducing CA19-9 and traditional proteins among PC serum biomarkers. In addition to seeing the landscape of current potential valuable biomarkers in serum-based early-stage PC diagnosis, we include a brief introduction to all single and multiplex novel serum biomarkers.

### 2.1. Proteins and Glycans

Alterations in serum protein or glycan levels demonstrate significant associations with disease progression, establishing their utility as candidate biomarkers. For instance, CA19-9 serum concentrations exhibit a positive correlation with tumor size in Lewis-positive PC patients [14]. Among the glycan and protein serum biomarkers, CA19-9, CEA, mucins, and osteopontin are representative in cancer diagnosis, especially in PC, breast cancer, and ovarian cancer. The subsequent section will provide a comprehensive summary of these biomarkers in PC diagnosis, with a specific focus on their potential applications in early detection.

#### 2.1.1. CA19-9

CA19-9, also known as sialyl Lewis A, is a tetrasaccharide structure identified by monoclonal antibody (1116-NS-19) [15,16,17,18]. As a glycan, it is decorated on diverse glycoproteins [19,20,21], including MUC1, MUC5AC, MUC16, CD44. It is associated with tumor hematogenous metastasis [22,23,24], oncogenic alteration [19,25] and immunosuppressive activity [26]. Clinically, CA19-9 is the only current approved serum biomarker for pancreatic cancer monitoring by the Food and Drug Administration (FDA) [27]. Meta-analytical data indicate that CA19-9 has 71–81% sensitivity and 82–90% selectivity in meta-analysis at a 37 U/mL threshold for distinguishing PC patients from healthy controls [28,29]. When the level of CA19-9 is >37 U/mL, it is associated with invasive intraductal papillary mucinous neoplasms (IPMN) [30]. CA19-9 also aids in differentiating pancreatic neuroendocrine carcinoma (PNEC) and pancreatic ductal adenocarcinoma (PDAC), which cannot be solved by imaging [31].

CA19-9 is not a universal biomarker for PC. CA19-9 levels rise proportionally with tumor burden in the Lewis antigen-positive population [14]. In contrast, due to the lack of the FUT3 enzyme, CA19-9 in both healthy people and pancreatic cancer patients is low in the Lewis antigen-negative population [19]. Furthermore, CA19-9 is non-tumor-specific. A high level of CA19-9 exists in the serum of patients with diverse types of cancer and inflammatory disease, such as pancreatitis, lung fibrosis, cirrhosis, hepatitis, liver cancer, lung cancer, and colon cancer [32]. These reduce the diagnostic specificity of CA19-9.

Despite suboptimal standalone performance, CA19-9 has increasing evidence to be a predictor for pancreatic cancer before clinical presentation [33]. For example, O’Brien et al. reported the first study in measuring serum CA19-9, CA125, CEACAM1, and REG3A levels in pre-diagnostic samples [34]. In this study, CA19-9 has 53% or 68% sensitivity at 95% specificity for predicting the clinical presentation of PC in 24 months or 12 months in advance. Combining CA125 with CA19-9 can increase the sensitivity of detection, since CA125 is elevated in Lewis-antigen negative patients. Similarly, Honda et al. used CA19-9 alone, achieving 50% sensitivity and 98% specificity for PDAC prediction 6 months ahead [35]. Combining with apolipoprotein-A2 isoforms, the prediction sensitivity is slightly improved to 57% at 98% specificity. This is consistent with the study performed by Fahrmann et al. and Mason et al. [36,37]. At 99% specificity, CA19-9 has 60% sensitivity up to 6 months prediction of PDAC diagnosis and 50% sensitivity for resectable pancreatic cancer cases [36]. For the cohort with low CA19-9 levels, a combination of CA19-9, LRG1, and TIMP1 gained 13.2% sensitivity for PDAC prediction up to 12 months [36]. Moreover, a longitudinal study by Fahrmann et al. further validated the prediction value of CA19-9 [38]. The continuous monitoring of CA 19-9 in pre-diagnostic blood samples improves the model sensitivity and lead-time detection of PDAC prediction. In low CA19-9 cohorts, TIMP1 further helps the sensitivity improvement in PDAC prediction. Except for predicting the clinical presentation of PC, CA19-9 is significantly associated with early recurrence in CA19-9-positive patients before imaging detection [39,40]. A 2.45-fold elevation of CA19-9 after surgery has 90% sensitivity and 83.33% specificity to predict PC recurrence [41]. These studies suggest that continuous measurement of CA19-9 in high-risk populations or in PC patients after surgery can take the lead in early diagnosis of PC.

In summary, CA19-9 itself is not recommended for screening [42,43], but it is still one of the strongest potential members in biomarker panels for diagnosing early-stage pancreatic cancer.

#### 2.1.2. CEA

CEA, also known as CEACAM5 or CD66e, is a heavily glycosylated glycoprotein potentially featuring Lewis X and sialyl Lewis X motifs on asparagine residues [44]. It is involved in cancer cell adhesion, carcinogenesis, tumor cell proliferation, metastasis, and anticancer immunity [44,45]. Particularly, it is associated with gastric cancer peritoneal dissemination [46] and distant metastasis of pancreatic cancer [47]. In the clinic, elevated serum CEA levels occur in multiple malignancies and benign conditions, including pancreatic cancer, colon cancer, rectum cancer, lung cancer, uremia, and lung fibrosis [44]. CEA level is not associated with malignancy during the IPMN stage [30]. In blood-based PC diagnosis, CEA alone yields an average of 43% sensitivity and 82% specificity to distinguish PC from healthy controls [48], and has lower overall diagnostic accuracy than CA19-9 alone [49]. Compared to CA19-9 (19.1%), CEA obtained an advantage in sensitivity in overall Lewis-negative antigen PC patients (63.85%) and in stage I/II PC patients (60.9%) [50]. The diagnostic ability of CEA (cut-off value 7.0 ng/mL) is independent of CA 19-9 for the late stage of PC [51]. Combining CEA with CA19-9 improved the positive predictive values for late-stage PC patients [51] and increased overall specificity in distinguishing PC and non-PC patients [52]. In short, CEA has advantages in independence from CA19-9, potential diagnostic ability in Lewis-antigen negative PC patients, and outperformance in early-stage PC patients compared to CA19-9. While CEA is insufficient as a standalone screening tool, it can serve as an important complementary biomarker for CA19-9 in early-stage PC patients.

#### 2.1.3. MUCINs (Mucin 1/CA15-3, Mucin 5AC, Mucin 16/CA125)

Mucins are a family of high-molecular-weight glycoproteins characterized by extensive O-glycosylation of their tandem repeat domains [53]. The mucin family comprises 21 members, categorized into transmembrane and secreted subtypes. In PC diagnostics, circulating biomarkers such as MUC1, MUC16, and MUC5AC have gained attention for their diagnostic potential in blood-based assays. Notably, while MUC1 and MUC16 are transmembrane mucins, their proteolytically cleaved extracellular domains, CA15-3 (a soluble form of MUC1) and CA125 (a soluble epitope of MUC16), serve as soluble biomarkers detectable in the serum of PC patients.

MUC1 is a transmembrane mucin with heavy O-glycosylation in normal cells [54]. It consists of three domains, including variable number tandem repeat (VNTR) region, sea urchin sperm protein enterokinase and agrin (SEA), and cytoplasmic tails (CT) [55]. The VNTR domain regulates tumor cell migration and metastasis, while the CT domain controls cell motility, apoptosis, and proliferation. In tumor-associated MUC1, the VNTR region is under-glycosylated, exposing the repeat peptide chain and serving as a potential target for distinguishing between normal and tumor cells [54]. In the clinic, MUC1 has been widely investigated in breast cancer but is also abnormally overexpressed in various types of cancer, such as lung, pancreatic, prostate, and colon cancers [54]. In PC, 77% of sensitivity and 95% of specificity for detecting PC at a serum cut-off level of 10.2 U/mL [56]. Furthermore, PAM4-reactive MUC1 was detected in 87% of early PC [57]. Unlike CA19-9, MUC 16, and CEA, level of MUC1 does not affect by pregnancy [58].

MUC16 is a transmembrane mucin with the CA125 epitope on the epithelial cell surface [59]. It promotes cancer cell proliferation by inhibiting apoptosis [60], facilitates cancer cell adherence and metastasis [61], and is associated with tumor immune evasion [62,63]. In PC, MUC16 enhances the PDAC metastasis via MMP-7 activation [64], supports progression and aggressive subtypes by modulating oncogenic signaling [65], and associates with tumorigenesis [66]. Via phosphorylation, CA125 is released from MUC16 and secreted into the serum [67]. In the clinic, an abnormal level of CA125 has been detected in pancreatic cancer [49,68,69,70], ovarian cancer [70,71], colorectal cancer [70,72] and biliary tract carcinoma [69,73]. In PC patients, the CA125 level is higher in patients with metastasis [50,74], yielding 59% sensitivity and 78% specificity for distinguishing PC patients from non-PC individuals [52]. Compared to CA19-9, CA125 is superior in predicting resectable PC patients [75], and has a higher expression level in Lewis antigen-negative patient tissue and serum [76]. CA125 has greater sensitivity (51.1%) than CA19-9 (19.1%) in Lewis antigen-negative PC patients [50], but it is lower in overall PC diagnostic accuracy [49]. Similar to CA19-9, CA125 has potential in differentiating PNEC and PDAC, which cannot be distinguished by imaging [31]. Combining CA125 with CA19-9 increased the overall performance for the diagnosis of PC [49,52,77,78], and improved 20% sensitivity in pre-diagnosis for low CA19-9 patients [34].

MUC5AC belongs to the secreted subtypes of mucins. It promotes PC stemness by potentiating oncogenic signaling (integrin β5/pSrc/pSTAT3 signaling) [79], inhibits PC cell apoptosis via TNF-related apoptosis-inducing ligand [80], and is associated with invasion and adhesion of PC by promoting the expression of integrins, MMP-3, VEGF and activating Erk pathway [81]. MUC5AC has been validated as a potential pancreatic disease biomarker in tissue in independent studies [82,83,84]. Recently, MUC5AC gained attention in cancer diagnosis in serum samples. In blood tests, MUC5AC has been detected in colon and pancreas cancer [85,86]. For pancreatic disease, differential expressions of MUC5AC are observed in patients with benign pathologies, chronic pancreatitis, and PC [87,88]. The level of MUC5AC is associated with predicting the recurrence in PDAC patients on neoadjuvant chemotherapy before resection [89]. MUC5AC has been investigated in PC detection in blood with 174.6 ng/mL in early pancreatic cancer and 228.7 ng/mL in late pancreatic cancer [87]. In serum-based PC diagnosis, MUC5AC alone yields 54.1% sensitivity and 95.1% specificity to distinguish PC from healthy controls [87]. In another study, it yields 83% sensitivity and 80% specificity to identify resectable early-stage PC from healthy controls [88]. In both studies, MUC5AC has been examined as a complement for CA19-9. When combined with CA19-9, MUC5AC improves diagnostic performance in both Caucasian and Asian populations [87,88]. Furthermore, MUC5AC on circulating extracellular vesicles has favorable sensitivity (82%) and specificity (100%) to distinguish high-grade invasive IPMN from low-grade IPMN [90]. Although a larger cohort study will validate the performance of MUC5AC in PC diagnosis, it highly indicates that MUC5AC has the potential to be an early-stage PC diagnosis biomarker.

In summary, despite mucins generally exhibiting lower sensitivity or specificity than CA19-9 in the overall population, they serve as important complementary biomarkers. Mucins can address the limitations of CA19-9 in Lewis antigen-negative populations and enhance diagnostic efficiency in early-stage PC patients. Their potential as early-stage PC biomarkers warrants further investigation in cross-validation.

#### 2.1.4. Osteopontin

Osteopontin is an N-linked secreted glycosylated, phosphorylated protein existing in mammalian body fluid [91]. It is overexpressed in various types of cancers, including pancreatic adenocarcinoma, colon, breast, lung, and ovarian tumors. This overexpression enhances tumor cell motility and angiogenesis by inducing COX-2 and PGE2 secretion. Furthermore, it inhibits cell apoptosis by decreasing the expression of anti-survival genes, and regulates tumor growth and invasion via ERK2 activation [92].

Osteopontin has been evaluated as a potential biomarker for pancreatic cancer [93,94]. Serum levels in pancreatic cancer patients are approximately 1.5 times higher than those in patients with lung, breast, and ovarian cancer. Notably, osteopontin levels do not correlate with tumor size, contrasting with CA19-9. Healthy individuals exhibit lower osteopontin levels (204 ± 65 ng/mL) compared to resectable pancreatic cancer patients (482 ± 170 ng/mL) [93]. At a cutoff of 334 ng/mL, osteopontin achieves 97% specificity and 80% sensitivity for diagnosing resectable pancreatic cancer, with sensitivity comparable to CA19-9 at its respective cutoff (37 U/mL) [93]. Osteopontin elevation is not tumor-specific, occurring in inflammatory conditions such as chronic granulomatous disease [93]. To address this limitation, studies have explored combining osteopontin with CA19-9 and metalloproteinase 1 (TIMP-1) for pancreatic cancer detection [95]. Osteopontin has been shown to outperform CA19-9 in distinguishing intraductal papillary mucinous neoplasms (IPMN) from chronic pancreatitis. Osteopontin level in PC patients (77.6 ± 67.3 ng/mL) is significantly different from that of healthy individuals (39.5 ± 32.8 ng/mL) and chronic pancreatitis patients (41.8 ± 29.8 ng/mL) in statistics [93]. This significant difference exists between resectable pancreatic cancer patients (62.4 ± 50.9 ng/mL) and healthy individuals as well, which is consistent with the previous study of Koopmann et al. [93]. Li et al. concluded that the osteopontin level in pancreatic cancer patients is significantly elevated compared to healthy individuals as well [94]. Similarly, Song et al. found that osteopontin outperforms CA19-9 in separating IPMN from chronic pancreatitis [96]. However, there is no noticeable difference in the serum level of Osteopontin between chronic pancreatitis patients and healthy individuals. This is consistent with the investigation of Rychlíková et al. but opposite to the result of Koopmann et al. [93,97]. To conclude, osteopontin is valuable as a complementary biomarker to CA19-9 for early-stage pancreatic cancer diagnosis. Its ability to differentiate chronic pancreatitis from healthy individuals highlights its potential utility in improving diagnostic accuracy when used in combination with other biomarkers. Despite its non-specific elevation in inflammatory conditions, the distinct serum levels of osteopontin in PC patients make it a promising adjunct in biomarker panels for enhanced diagnostic performance.

### 2.2. Extracellular Vehicles (EVs) and Circulating Tumor Cells (CTCs)

Extracellular vehicles (EVs), consisting of exosomes and microvesicles, are released by both cancerous and non-cancerous cells. They contain a multitude of biomolecules, such as proteins, nucleic acids, and metabolites within a lipid bilayer membrane [98,99]. The composition of EVs reflects the metabolic and disease state of their parent cells [100,101], making them promising biomarkers for liquid biopsy diagnostics. Several proteins and nucleic acids on EVs have been investigated as biomarkers for PC (Table 1). For instance, a sole marker, Glypican-1 (GPC-1) on pancreatic cancer exosomes has been reported as a marker for detecting early-stage PC with 100% sensitivity and specificity from healthy controls [102]. GPC-1-positive exosomes recently also show potential in distinguishing PDAC patients from chronic pancreatitis (CP) patients, achieving an AUC of 0.974 for stage I PDAC detection [103]. However, the sensitivity and specificity of GPC-1-positive exosomes vary with detection methods. It yields 80.0% sensitivity and 93.3% specificity by using an immunoassay based on hierarchical surface-enhanced Raman scattering substrate in distinguishing PDAC and healthy controls [104]. However, after combining with LRG1 protein on exosomes, good sensitivity (90%) and specificity (86.7%) are still obtained to distinguish early-stage PC patients from healthy controls [104]. Additionally, combining GPRC5C-positive exosomes and EPS8-positive exosomes can even detect early-stage PDAC patients with low CA19-9 levels [98]. Additionally, the combination of multiple protein-positive exosomes can achieve tumor specificity. A panel of Clathrin Heavy Chain (CLTC), Ezrin (EZR), Talin-1 (TLN1), Adenylyl cyclase-associated protein 1 (CAP1), and Moesin (MSN) positive exosomes demonstrated 0.94–0.99 accuracy against other cancers [105]. MicroRNAs (miRNAs) within EVs also exhibit high sensitivity and specificity in differentiating PDAC patients from healthy and pancreatic benign disease patients. For example, miR-45 Ia-positive exosomes are significantly associated with PC stage and distant metastasis, showing favorable AUCs in distinguishing PC patients from both healthy (0.896) and benign disease patients (0.855) [106].

In addition to EVs, CTCs, the intact cells originating from a specific solid tumor, are potential disease biomarkers for pancreatic tumors, given that they can be identified in the peripheral blood even prior to the onset of PC metastasis [107]. For example, most recently, IL-10R2+/IL-22R1+ myeloid cells in peripheral blood have been identified as a potential predictive marker for PDAC recurrence [108]. In diagnosis, an average 65% detection rate is present in pancreatic cancer CTC detection [109]. Although the low number of CTCs in PDAC compared with other epithelial cancers makes detection challenging [110], sensitive and specific CTCs detection techniques are gradually being developed. Ankeny developed a method combining the NanoVelcro platform with high-resolution fluorescent microscopy and multi-color immunocytochemistry, successfully achieving 75% sensitivity and 96.4% specificity in detecting pancreatic CTCs [111]. Vimentin-positive CTCs detected by a microfluidic chip-based platform can distinguish 65% of PDAC patients from healthy controls with 100% specificity under a cutoff of 2 cells/4mL. Although it is inferior to CA19-9 as a sole biomarker in sensitivity, combining vimentin-positive CTCs with CA19-9 improves overall diagnostic performance to an AUC of 0.968 [112].

**Table 1:** Serum-based biomarkers for differentiating early-stage or all-stage pancreatic cancer patients from pancreatic benign disease or healthy donors. Molecules are listed with AUC larger than 0.75. The order within each group is followed by individuals and then multiplex panels. AUC: area under the curve; SN: sensitivity; SP: specificity; PC: pancreatic cancer; BPD: benign periampullary disease; BP: benign pancreatic cystic neoplasms; PB: pancreatic benign disease; dCCA: distal cholangiocarcinoma; CPA: serum carboxypeptidase A; PHA-E-positive Cp: Phaseolus vulgaris Erythroagglutinin-Positive Ceruloplasmin; MMP-7: Matrix metalloproteinase-7; SDC1: Syndecan-1; LTB4: leukotriene B4; sCD40: soluble CD40; sCD163: soluble CD163; GLRX3: Glutaredoxin3; GPC-1: Glypican-1; PRKCB: protein kinase C beta type gene; 5hmC: 5-hydroxymethylcytosine; qRT-PCR: Quantitative reverse transcription and real-time polymerase chain reaction; NGS: Next-Generation Sequencing; CLIA: chemiluminescent immunoassay; ECLIA: electrochemiluminescence immunoassay; TELQAS: target enrichment long-probe quantitative amplified signal assay.

Biomarker Names	Disease Stage	AUC	SN	SP	Detection Method	Ref.
**Glycans and proteins**						
sCD163	Early-stage PC vs. health	0.93	-	-	ELISA	[113]
PHA-E-positive Cp	PDAC vs. health	0.97	-	-	MS and Lectin blotting	[114]
fibrinogen alpha chain	PDAC vs. health	-	67.4%	83.6%	MS	[115]
serum N-glycome	PDAC vs. health	0.81	75%	72%	MS	[116]
TNF-α, IL-2R, IL-6, and IL-8	malignant IPMNs vs. benign IPMN	0.87	88.7%	73.1%	CLIA	[117]
CA19-9 + GLRX3	PDAC vs. health	-	98.3%	100%	ELISA	[118]
CA19-9 + bilirubin	Early-stage PDAC vs. BPD	0.89	-	-	Immunoassay, diazo method, Luminescence,	[119]
	CA19-9 < 37 U/mL patient:	0.84	-	-		[119]
CA199 + PIVKA-II	PDAC vs. PB	0.95	83.3%	94.4%	CLIA	[120]
CA19-9 + LTB4	PDAC vs. health	0.97	-	-	ELISA	[121]
CA19-9 + sCD40	Early-stage PC vs. health	0.95	-	-	ELISA	[122]
CA19-9 + MMP-7 + SDC1	PDAC vs. health	1.00	100%	33%	Immunoassay	[123]
CA19-9 + MMP-7	PDAC vs. health	0.99	99%	65%	Immunoassay	[123]
CA19-9 + sAXL	PDAC vs. CP	-	89.9%	100%	ELISA	[124]
	PDAC vs. controls	0.91	100%	-		[124]
CA19-9 + CA19-9/bilirubin ratio +bilirubin	PDAC vs. PB	0.91	90%	80%	Immunoassay, diazo method, Luminescence	[125]
	PDAC vs. dCCA	0.83	64.6%	90.9%		[125]
CA19-9 + Asprosin	early stage PDAC vs. diabetics patient	0.93	-	-	ELISA	[126]
CA19-9 + MMP2	PC stage I vs. health	-	85%	96%	PAC-MANN assay, ELISA	[127]
EphA2-NF + CA 19-9	Early-stage PC vs. health	0.94	-	-	CLIA	[128]
**Circulating Tumor Cells**						
CA19-9 + CD86, CA14, CD33, CD4, CD11b, CD183, CD25, CD152 and 11 cell subsets	Early-stage PC vs. health	0.95	-	-	MS	[129]
	PDAC vs. health	0.98	92.4%	91.2%		[129]
**Extracellular Vesicles (detection marker)**						
CD63	PDAC vs. Health	0.85	82.1%	84.6%	ELISA	[130]
GPRC5C + EPS8	Early-stage PC vs. health	0.92–0.95	-	-	Ultracentrifugation, MS	[98]
hsa_circ_0006220 + hsa_circ_0001666	PDAC vs. Health	0.88	74.2%	87.1%	qRT-PCR	[131]
GPC-1	stage I PDAC vs. CP	0.97	-	-	flow cytometry	[103]
miR-45 Ia	PC vs. health	0.90	80.1%	86.7%	qRT-PCR	[106]
	PC vs. PB	0.86	71.2%	89.5%		[106]
MUC5AC	invasive high-grade IPMN vs low-grade-IPMN	0.96	82%	100%	flow cytometry	[90]
LRG1 + GPC-1	Early-stage PC vs. health	0.95	90.0%	86.7%	Immunoassay	[104]
CA19-9 + GPC-1 + CD82	PDAC vs. CP	0.96	-	-	flow cytometry	[132]
	PDAC vs. health	0.94	-	-		[132]
**microRNAs and tsRNAs**						
miR-320b	PDAC vs. CP	1	100%	100%	qRT-PCR	[133]
miR-122-5p	PDAC vs. health	0.99	98%	96%	qRT-PCR	[133]
hsa-miR-1246+hsa-miR-205-5p+ hsa-miR-191-5p	PDAC vs. CP	0.92	94.5%	80%	machine learning	[134]
CA19-9 + miR-34a-5p + miR-130a-3p + miR-222-3p	PDAC stage II vs. controls	0.92–0.94	-	-	miRNA profiling platform	[135]
CA19-9 + miR-1290	PDAC vs. health	0.963	80.4%	90%	miRNA sequencing	[136]
CA19-9 + 100 highly expressed miRNAs	asymptomatic early-stage PC vs health	0.97	67%	98%	miRNA sequencing, machine learning	[137]
	PC vs. health	0.99	90%	98%		
tRF-Pro-AGG-004 + tRF-Leu-CAG-002	PDAC vs. health	-	85%	96.4%	RNA sequencing	[138]
	Early-stage PC vs. health	0.84	75%	83.0%		[138]
**ctDNAs and cfDNAs**						
hypermethylation of BMP3, RASSF1A, BNC1, MESTv2, TFPI2, APC, SFRP1 and SFRP2	PDAC all stages vs. CP	0.85–0.93	-	-	PCR + CLIA.	[139]
5hmC densities in cfDNAs	PDAC vs. health	0.92–0.94	-	-	NGS	[140]
six DNA methylations of PRKCB	PDAC vs. CP	1	-	-	Bisulfite qPCR	[141]
cfDNA-derived 5-hydroxymethylcytosine (5hmC)	Early-stage PC vs. high-risk populations	-	68.3%	96.9%	NGS + machine learning	[142]
CA19-9 + FUT3 test	PDAC (localized) vs. controls	-	66.4%	99.3%	ELISA + gene sequencing	[143]
CA19-9 + thirteen methylated DNA markers (AK055957, GRIN2D, CD1D, ZNF781, FER1L4, RYR2, CLEC11A, LRRC4, GH05J042948, HOXA1, PRKCB, SHISA9, NTRK3)	Early-stage PC vs. health	0.9	81%	97.5%	ELISA + TELQAS	[144]
	PDAC vs. health	-	92%	92%		[144]
CA19-9 + 56-marker classifier	Early-stage PC vs. health	0.92	88%	89%	bisulfite sequencing	[145]
	PDAC vs. health	0.94	86%	89%		[145]
CA19-9 + DUPAN-2 + FUT test	PC vs. health	-	80.7%	97.7%	ELISA + gene sequencing	[146]
	Early-stage PC vs. health	-	60.4%	97.7%		[146]
**Lipids and metabolites**						
Proline, creatine, and palmitic acid	early-stage PDAC vs. BP	0.852	-	-	MS	[147]
CA19-9 + Proline, creatine, and palmitic acid	early-stage PDAC vs. BP	0.91	-	-	MS	[147]
CA19-9 + four metabolites	Early-stage PC vs. health	0.76–0.85	39.7–81.6%	88.7–94.1%	MS + machine learning	[148]
**Mixed panels**						
Five proteins (EEF1A1, RPH3AL, NCOR1, L1CAM, TMEM161A) and three miRNAs (miR-146a-5p, miR-155–5p, miR-375)	high-risk IPMN vs. low-risk IPMN	0.97	-	-	qPCR + microarrays	[149]
SNP-stratified [CPA activity + CA19-9]	PDAC all stages vs. health	0.94	68%	98.2%	enzymatic assays, ELISA, TaqMan Assay	[150]
	PDAC stage I vs. health	0.90	51.9%	98.2%		[150]
CA19-9 + EV-CK18 mRNA + EV-CD63 mRNA + EV-miR.409 + cfDNA concentration	PDAC vs. health	0.95	88%	95%	PCR+ ECLIA + machine learning	[151]

### 2.3. Nucleic Acids (Circulating RNAs and DNAs)

MicroRNAs (miRNAs) are small non-coding RNAs with approximately 19–23 nucleotides existing in various biological fluids [136]. They have the potential to be pancreatic tumor diagnostic biomarkers due to their roles in promoting tumor growth, tumor metastasis, and chemoresistance [152,153,154]. Recently, several miRNAs have been discovered as pancreatic cancer biomarkers in serum (Table 1). For instance, miR-122-5p performs well in distinguishing PDAC patients from healthy individuals with 98% sensitivity and 96% specificity [133]. A panel of three miRNAs (hsa-miR-1246, hsa-miR-205-5p, and hsa-miR-191-5p) yields 94.5% sensitivity and 80% specificity in distinguishing PDAC patients from CP patients [134]. A meta-analysis reported that miRNA achieves an average of 79% sensitivity and 77% specificity with AUCs of 0.85 for PC [155]. For early-stage PC, miRNA obtains average sensitivity and specificity of 79% and 74%, respectively, with AUCs of 0.81.

tRNA-derived small RNAs (tsRNAs) are small non-coding RNAs generated from tRNAs by specialized nucleases and may have functional similarity to miRNA [156,157]. tsRNAs are potential pancreatic tumor diagnosis biomarkers playing roles in tumorigenesis and tumor progression that promote cell invasion and metastasis in pancreatic cancer [138,156]. The combination of tRF-Pro-AGG-004 and tRF-Leu-CAG-002 has been recently discovered to surpass commonly used clinical markers like CA19-9 and CEA in the diagnosis of PDAC or early-stage PDAC patients from healthy individuals [138]. This combination shows high specificity (96.4%) and moderate sensitivity (85%) in distinguishing PDAC patients and healthy individuals, and moderate sensitivity (75%) and specificity (83%) in identifying early-stage PDAC patients from healthy controls.

Circulating tumor DNA (ctDNA) derives from tumor cells, and circulating cell-free DNA (cfDNA) derives from both normal and tumor cells. The concentration of ctDNA and cfDNA is associated with stages of tumor and metastatic burden, making them tumor biomarkers [158]. DNA mutations and DNA methylations are key alternatives in circulating DNA tumor biomarkers (Table 1). For example, for ctDNA mutation, Kirsten Rat Sarcoma (KRAS) mutation has potential in diagnosing PDAC within a healthy population, with good specificity (82–100%) but variable sensitivity (21–86%) [159]. For ctDNA methylation, a panel of six methylation sites on the protein kinase C beta type gene (*PRKCB)* demonstrates perfect distinction between PDAC patients and CP patients (AUC = 1.0) [141]. Additionally, cfDNA-derived 5-hydroxymethylcytosine (5hmC) densities help identify PDAC patients from healthy individuals with good AUC (0.92–0.94) [140]. Furthermore, cfDNA-derived 5hms shows high specificity (96.9%) with moderate sensitivity (68.3%) in distinguishing between early-stage PC patients from non-cancer high-risk populations [142].

### 2.4. Lipids and Metabolites

Lipid metabolism is influenced by oncogenic KRAS in pancreatic cancer, promoting studies into lipidomic profiling in pancreatic cancer diagnosis [160,161]. Recently, a panel of lipid profiles has been reported to have high ability (97.4% sensitivity and 97.4% specificity) to distinguish early-stage PDAC patients from healthy controls, outperforming CA19-9 [161]. Combining the lipid profiles with CA19-9 can further enhance specificity.

Metabolic reprogramming during the process of normal to tumor cells leads to metabolites as potential diagnostic biomarkers for PDAC [147]. A panel with proline, creatine, and palmitic acid has the ability to identify early-stage PDAC patients from benign pancreatic cystic neoplasms patients (BP) (AUC = 0.852) [147]. When combined with CA19-9, diagnostic performance increases to an AUC of 0.909. This combination also robustly distinguishes early-stage PDAC patients from healthy individuals with an AUC of 0.949, showing potential to be an early diagnosis of PC [147].

### 2.5. Multiplex Biomarker Panels

To date, there is no single golden biomarker for screening pancreatic cancer patients. Although CA19-9 is the only FDA-approved biomarker for pancreatic cancer, it lacks efficacy as a standalone screening tool [6]. Multiplex biomarker panels, which combine multiple markers to compensate for individual limitations, offer improved diagnostic performance for identifying PDAC or early-stage PDAC patients from healthy individuals or those with pancreatic benign diseases. Recently discovered multiplex biomarker panels are included in Table 1. Since researchers have been increasingly focusing on combining CA19-9 with multiple serum biomarkers to improve the overall diagnostic performance of CA19-9 for pancreatic cancer diagnosis, recent multiplex panels often include CA19-9. In the current section, we will focus on the biomarker panels with CA19-9 for identifying early-stage pancreatic cancer.

It has been demonstrated that panels with CA19-9 and protein biomarkers can discriminate early-stage pancreatic cancer patients from healthy individuals, or early-stage pancreatic cancer patients from pancreatic benign disease, even in cases with low CA19-9 levels. Matrix metalloproteinase-2 (MMP-2), involved in PDAC progression, achieves favorable specificity (96%) and moderate sensitivity (85%) when combined with CA19-9 for identifying stage I PDAC patients from healthy controls [127]. The protease activity of MMP-2 is utilized in the non-invasive PAC-MANN assay [127]. When combined with ELISA, early-stage PC patients were detected from healthy controls. Erythropoietin-producing hepatocellular ephrin receptor A2 N-terminal fragment (EphA2-NF), independent of CA19-9, demonstrates good AUCs (0.94) in identifying early-stage PDAC patients from healthy controls [128]. SolubleCD40 (sCD40), which increases regardless of PDAC stage and outperforms CA19-9 in IPMN stage, enhances the AUC (0.945) for resectable PDAC when combined with CA19-9. In diabetes patients, a high-risk population for PC, combining CA19-9 with asprosin yields a perfect AUC of 0.925 for identifying early-stage PC patients [126]. For low CA19-9 level PC patients, combining CA19-9 with bilirubin detects early-stage PC patients from benign periampullary disease with an AUC of 0.84 [119].

Combining CA19-9 with nucleic acids or gene tests also improves diagnostic performance in distinguishing early-stage pancreatic cancer from healthy individuals. CA19-9 coupled with highly expressed miRNAs yields favorable specificity (98%) and AUC (0.97) with moderate sensitivity (67%) for detecting asymptomatic early-stage PDAC patients from healthy controls [135]. When CA19-9 is paired with thirteen methylated ctDNA, high specificity (97.5%) and moderate sensitivity (81%) are achieved in identifying stage I-II PC patients from healthy individuals.

Given that CA19-9 biosynthesis is mediated by genetic polymorphisms of the FUT2 and FUT3 enzymes [143,162,163], genetic stratification based on single-nucleotide polymorphisms (SNPs) offers a viable strategy for enhancing CA19-9 sensitivity and specificity in early PC diagnosis. For example, Luo et al. have divided over 1000 cohorts into low, medium, and high CA19-9 biosynthesis groups based on Lewis (FUT3) and secretor (FUT2) genotyping [164]. In medium and high CA19-9 biosynthesis groups, the sensitivity for CA19-9 in detecting stage I and stage II of PC increases from 76.1% to 87.2%, with 92.2% specificity. In the Lewis antigen-negative PC patients, CA19-9 is not ideal but still useful for detecting PC with 48.6% sensitivity and 95.9% specificity, by adjusting the cut-off value from 37.0 U/mL to 1.8 U/mL. Consistently, Abe et al. demonstrated a four-group classification based on genetic subgroups of FUT3 and FUT2 [143]. The model yields an overall performance with 60.8% sensitivity and 98.8% specificity by CA19-9 for distinguishing PDAC patients from healthy controls. Based on the four-group classification, Dbouk et al. further developed a CA19-9 tumor marker gene test with personalized individual’s CA19-9 reference range [165]. The overall diagnostic sensitivity and specificity for identifying PDAC patients from healthy individuals are 67.2% and 91.4%, respectively. Of note, for distinguishing stage I PDAC from healthy controls, this test displays 46.4% sensitivity with 98.9% specificity. Ando et al. further introduced serum DUPAN-2 as a biomarker for individuals unable to synthesize CA19-9 [146]. Based on CA19-9 and DUPAN-2 functions in different FUT2 and FUT3 variants, individuals are assigned to five groups. The combination test of FUT/CA19-9 and FUT/DUPAN-2 performs improved sensitivity (78.4%) at 97.7% specificity for identifying stage I and stage II PDAC from controls.

In summary, CA19-9 remains a pivotal anchor among PC serum biomarkers. Its potential for early-stage pancreatic cancer screening is enhanced when combined with complementary proteins, EVs, miRNAs, ctDNAs, lipids, metabolites, or FUT gene tests. Multiplex biomarker panels are crucial for achieving favorable diagnostic performance in early-stage PC patient diagnosis. By integrating CA19-9 with other biomarkers and leveraging genetic stratification, the diagnostic efficacy of CA19-9 can be significantly improved, offering new avenues for early detection strategies.

## 3. Aptamer-Based Biosensor

The precise recognition of pancreatic cancer biomarkers is pivotal for early diagnosis and dynamic monitoring. Building upon the biomarker spectrum discussed previously (e.g., CA19-9, CEA, MUC1, CA125, osteopontin), aptamers—single-stranded oligonucleotides with high specificity, programmability, and stability—have emerged as ideal molecular probes for biosensor development. Over the past decade, significant progress has been made in identifying aptamers targeting pancreatic cancer-associated biomarkers. After Systematic Evolution of Ligands by Exponential Enrichment (SELEX), the binding affinities of these aptamers have been rigorously validated using techniques such as surface plasmon resonance (SPR), isothermal titration calorimetry (ITC), microscale thermophoresis (MST), and fluorescence polarization (FP). Among the PC serum biomarkers (Table 2), CEA, MUC1, and CA125 are among the most investigated biomarkers in aptamer development. Exosomes are typical novel PC serum biomarkers that can be detected by aptamer. Additionally, CA19-9 is an important anchor among PC serum multiplex biomarkers. Hence, this section will mainly summarize the updated techniques of aptamer-based sensors, based on four specific PC serum biomarkers (CA19-9, CEA, MUC1, and CA125) and novel biomarker exosomes as well.

### 3.1. CA19-9 Detection via Aptamer-Based Biosensors

#### 3.1.1. Aptamer-Based Optical Biosensor

In aptamer-based sensing platforms, optical technologies have been widely applied in various fields, including environmental monitoring and medical diagnostics, due to their high sensitivity, excellent specificity, and intuitive detection capabilities. The core component of an optical biosensor is the transducer, which recognizes the specific interaction between biological recognition elements (such as aptamers) and target analytes and subsequently converts this biochemical recognition event into a detectable optical signal.

Currently, there are relatively few studies on aptamer-based detection of CA19-9, and research in this field is still in its exploratory stage. Among the published studies, two types of biosensors have demonstrated the potential of aptamers for CA19-9 detection: fluorescence-based sensors, which rely on fluorescence changes triggered by aptamer binding, and surface-enhanced Raman spectroscopy (SERS)-based sensors, which utilize Raman signal enhancement for detection. Although the number of related studies is limited, these methods have shown advantages in terms of high sensitivity.

##### Fluorescence-Based Aptamer Biosensor

**Figure 1:**
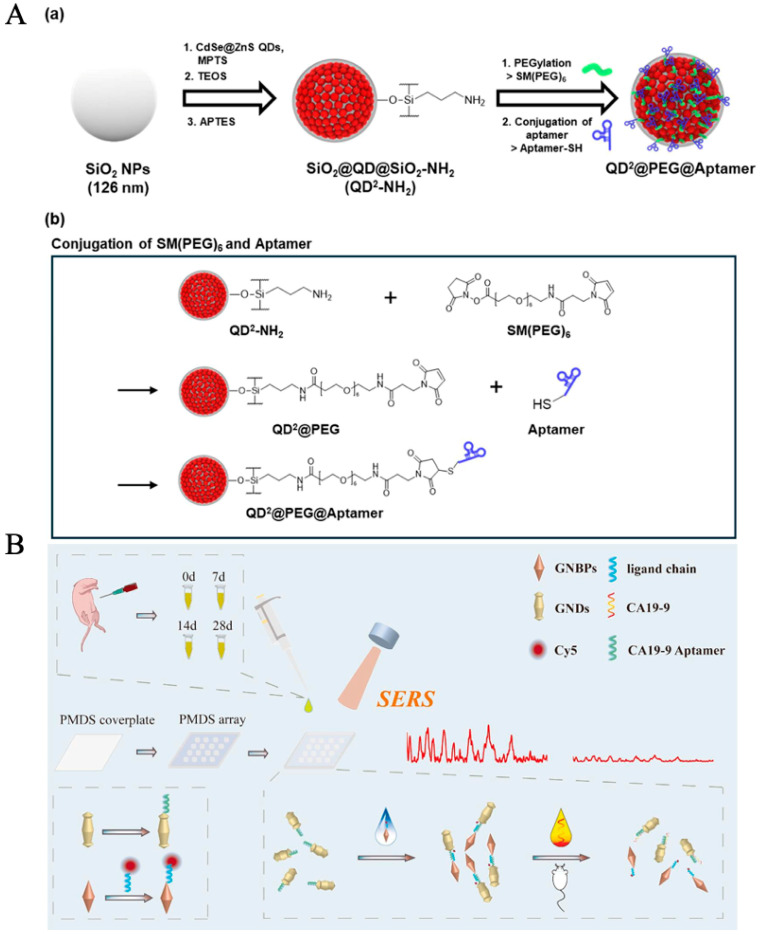
Comprehensive overview of aptamer-based biosensors for CA19-9 detection, focusing on optical biosensing techniques. (**A**): (**a**) Schematic of QD^2^@PEG@Aptamer fabrication. (**b**) Schematic of reaction process. Figure reproduced from [181], licensed under CC BY 4.0. (**B**). The schematic diagram for CA19-9 detection by the SERS aptasensor [182]. Copyright © 2024 Published by Elsevier B.V.

Yoo et al. developed a QD^2^@PEG@Aptamer nanoprobe-based biosensor for the detection of pancreatic cancer (PC) biomarker CA19-9, as shown in Figure 1A [181]. The nanoprobe consists of quantum dots (QDs) embedded in a silica (SiO_2_) core and surrounded by an outer SiO_2_ shell, exhibiting significantly enhanced fluorescence intensity (FI). By conjugating aptamers with QD^2^ nanoparticles, the research team constructed a lateral flow immunoassay (LFIA) system for highly sensitive detection of CA19-9. The system demonstrated a detection limit of 1.74 × 10^−2^ mg·mL^−1^, showing excellent selectivity and sensitivity. Experimental results indicated that the nanoprobe maintained stable fluorescence intensity for up to 10 days and exhibited high specificity for CA19-9, effectively distinguishing it from other biomarkers (e.g., Aβ40 and PSA). This study highlights the potential of QD^2^ and aptamer conjugation technology in diagnostic applications, particularly for the early detection of PC.

##### Surface-Enhanced Raman Spectroscopy-Based Aptamer Biosensor

Xia et al. developed a highly sensitive signal-off biosensor based on the SERS effect of gold nanodumbbells (GNDs) and gold nanobipyramids (GNBs), utilizing aptamers (“CA19-9 aptamer”, see Table 2) for the specific detection of CA19-9 as shown in Figure 1B [182]. Under optimized conditions, the sensor achieved a LOD of 1.16 × 10^−3^ U/mL and a limit of quantification (LOQ) of 3.87 × 10^−3^ U/mL. Furthermore, the sensor’s reliability in clinical samples was validated by measuring CA19-9 levels in mouse serum. Compared to the traditional enzyme-linked immunosorbent assay (ELISA), the sensor demonstrated high consistency, with relative errors (RE) ranging from 2.54% to 6.093%. This study highlights the potential of SERS-based aptamer sensors for cervical cancer screening and provides new insights for the detection of other diseases.

#### 3.1.2. Electrochemical-Based Aptamer Biosensor

Lin et al. developed a ratiometric electrochemical aptamer-based biosensor for the detection of CA19-9 in serum [183]. The sensor utilized AuNPs modified with reduced graphene oxide (rGO) and carboxylated multi-walled carbon nanotubes (cMWCNTs) as the sensing substrate. By labeling the CA19-9-specific aptamer (“CA19-9 aptamer”, see Table 2) with ferrocene (Fc) and its complementary strand with anthraquinone-2-carboxylic acid (AQ), a sensing interface with both specific recognition and amplified electrochemical signals was constructed. The redox reactions of Fc and AQ during the electrochemical process generated distinct electrical signals, with Fc oxidation producing the response signal (I_Fc_) at approximately 0.3 V and AQ oxidation providing the reference signal (I_AQ_) at approximately −0.2 V. The sensor enabled quantitative detection of CA19-9 by calculating the ratio of these two signals, denoted as I_Fc_/I_AQ_. Under optimized conditions, the logarithm of CA19-9 concentration exhibited a strong linear correlation with I_Fc_/I_AQ_, with a linear range spanning from 1.0 mU/mL to 1.0 × 10^6^ mU/mL and a detection limit of 0.65 mU/mL. The recovery rates of CA19-9 in serum samples ranged from 94.12% to 97.83%. Demonstrating excellent selectivity, reproducibility, and stability, this sensor offers a simple and effective strategy for the rapid and accurate detection of other tumor markers.

### 3.2. CEA Detection via Aptamer-Based Biosensors

#### 3.2.1. Aptamer-Based Optical Biosensor

In recent years, researchers have developed various optical biosensor platforms for CEA detection, including colorimetric sensors, fluorescence, chemiluminescence (CL), surface plasmon resonance (SPR), and electrochemiluminescence (ECL) biosensors. Each of these techniques offers distinct advantages, contributing to enhanced detection sensitivity, reduced analysis time, and label-free detection. In the following section, we will focus on the commonly used optical biosensors for CEA detection.

##### Colorimetric-Based Aptamer Biosensor

Colorimetric biosensors have gained significant attention among various optical detection methods due to their label-free nature, low cost, operational simplicity, portability, and the ability to enable visual detection of color changes with the naked eye [184,185]. The detection principle primarily relies on the specific interaction between the target molecule and the colorimetric probe, which induces a color change within the visible spectrum. Aptamer-based colorimetric biosensors mainly include gold nanoparticles (AuNPs) sensors [186] and peroxidase-mimicking sensors [187]. AuNPs are considered the most popular nanomaterials for aptasensor development due to their unique physical and chemical properties. AuNP-based sensors typically utilize the aggregation-dispersion transition of AuNPs, which alters their localized surface plasmon resonance (LSPR) properties, leading to a visible color change in the solution. For instance, in the absence of the target molecule, AuNPs remain dispersed, exhibiting a red color. However, when the target analyte (e.g., CEA) binds to the aptamer, it induces AuNP aggregation, resulting in a blue or purple color shift. Additionally, some AuNP-based sensors rely on the modulation of their peroxidase-like activity, where catalytic reactions produce colorimetric signals for detection. However, it is important to note that colorimetric assays based on AuNPs aggregation are susceptible to non-specific adsorption of the target onto nanoparticles, which can produce false-positive results. Such limitations have been previously reported [188]. DNAzyme sensors are another type, operating based on DNAzyme-catalyzed chromogenic reactions. Specifically, G-quadruplex DNA aptamers can catalyze the reaction of hydrogen peroxide (H_2_O_2_) with chromogenic substrates, leading to the production of colored compounds, thereby enabling quantitative or semi-quantitative detection of CEA. Due to their simple detection process, cost-effectiveness, and independence from expensive instrumentation, colorimetric biosensors are particularly suitable for on-site rapid detection and commercial applications, demonstrating great potential for CEA detection.

Luo et al. developed a simplified colorimetric biosensor for the detection of carcinoembryonic antigen (CEA) based on the conformational change of a CEA-specific single-stranded DNA aptamer (“b1-18 (5′ primer)”, see Table 2) and salt-induced aggregation of gold nanoparticles (AuNPs) [186]. Notably, the b1-18 aptamer [167], originally designed as a primer, shows the lowest reported Kd (0.69 nM) for CEA, which is a rare phenomenon in aptamer SELEX. It has also become the most widely used in CEA assays, with subsequent studies developing optimized derivatives (P-ATG and GAC-P) [169]. This method exploits the unique aggregation behavior of unmodified AuNPs, where the stability of AuNPs is regulated by the specific interaction between the aptamer and CEA. In the absence of CEA, the aptamer adsorbs onto the surface of AuNPs, maintaining them in a dispersed state, resulting in a red-colored solution. However, in the presence of CEA, the aptamer preferentially binds to the target molecule, causing the AuNPs to lose their protective coating and aggregate under high NaCl concentration, leading to a color change from red to blue. This color shift can be detected visually or analyzed spectroscopically. By optimizing the concentrations of the aptamer and NaCl, this method achieves a sensitive linear detection range for CEA (10–120 ng/mL) with a detection limit (LOD) as low as 3 ng/mL, making it adaptable to various detection requirements. Similarly, Liang et al. integrated hyperbranched rolling circle amplification (HRCA) with the same salt-induced AuNP aggregation mechanism [189]. In their design, CEA binding prevents the aptamer (“b1-18 (5′ primer)”, see Table 2) from hybridizing with complementary DNA (cDNA), allowing HRCA to generate single-stranded DNA (ss-DNA) that stabilizes AuNPs and maintains the red color. Without CEA, the aptamer binds to cDNA, blocking HRCA, leading to AuNP aggregation and a blue color change. Yang et al. developed a colorimetric aptasensing platform based on the principle that metal ions can enhance the peroxidase-like activity of AuNPs; the aptamer is b1-18 (5′ primer, see Table 2) [190]. The platform consists of a magnetic bead-supported sandwich aptamer capture module, a hybridization chain reaction (HCR) signal transduction module with tunable Hg^2+^ binding capacity, and a colorimetric detection module regulated by the peroxidase-like activity of AuNPs. By adjusting the Hg^2+^ binding capacity within the HCR system, the color mutation point can be tuned within the range of 4–25 ng/mL, enabling intuitive determination of the clinical threshold for CEA (5 ng/mL). This method achieved an LOD of 0.19 ng/mL and demonstrated 100% sensitivity and 96.67% specificity in actual serum samples, providing a novel strategy for convenient on-site detection of disease biomarkers.

**Figure 2:**
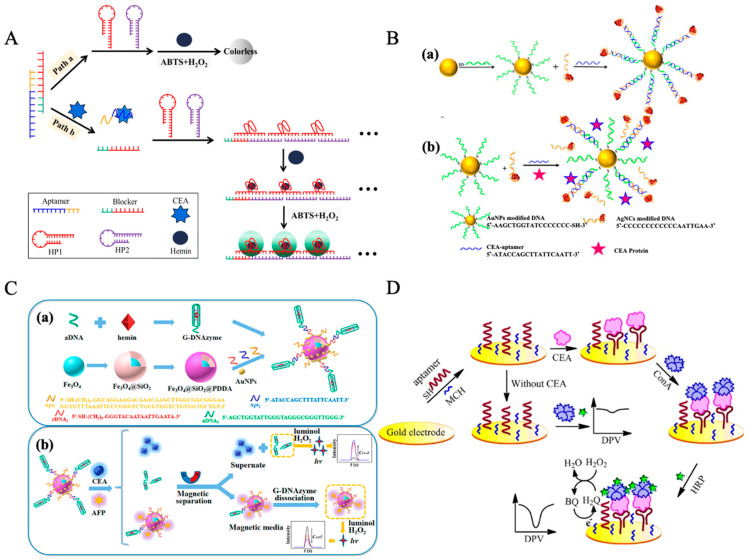
Aptamer-based biosensors for CEA detection, showcasing advancements in optical and biochemistry biosensor studies through signal amplification, fluorescence enhancement, chemiluminescence strategies, and biochemical methodologies. (**A**) Schematic illustration of CEA detection based on the dual signal amplification guaranteed by the coupling of HCR and the subsequent formation of hemin/G-quadruplexes with peroxidase-like activity [191]. Copyright © 2021 Wiley Periodicals LLC. (**B**): Schematic figure of CEA detection based on Surface-enhanced fluorescence (SEF). (**a**) Schematic illustration of SEF occurring. (**b**) Schematic illustration of the creative strategy for assaying CEA [193]. Copyright © 2014 Elsevier B.V. All rights reserved. (**C**): Schematic illustration of the Chemiluminescence aptasensor for CEA detection. (**a**) Preparation process diagram of the functional composite and (**b**) a schematic diagram of the CL sensor for detection of AFP and CEA based on the functional composite [203]. Copyright © 2023, American Chemical Society (**D**). Schematic illustration of the label-free and ConA-based sandwich aptasensor for CEA detection [211]. Copyright © 2017 Elsevier B.V. All rights reserved.

Shahbazi et al. developed a simple and rapid aptasensor based on split peroxidase DNAzyme for the visual detection of CEA [187]. By utilizing the target-induced reassembly of DNAzyme, this label-free colorimetric biosensor enables sensitive and specific CEA detection with a detection limit of 1 ng/mL. The method demonstrated high selectivity against non-target proteins and effective functionality in saliva, offering a promising, cost-effective strategy for non-invasive CEA screening in point-of-care applications. In addition to the split DNAzyme-based CEA detection method, Li et al. proposed a colorimetric detection strategy that integrates hybridization chain reaction (HCR) and hemin/G-quadruplex signal amplification, enabling a label-free and enzyme-free CEA assay as shown in Figure 2A [191]. This approach employs aptamer probes (“P-ATG”, see Table 2) for specific recognition, releasing a blocker sequence that triggers HCR, leading to the formation of peroxidase-mimicking hemin/G-quadruplexes, which catalyze the oxidation of ABTS and generate a visual signal. Although this method exhibits a relatively higher detection limit of 24.8 ng/mL and a longer detection time of 6 h, it demonstrates high selectivity by effectively distinguishing CEA from other proteins and achieves good recovery rates in serum samples ranging from 92.2% to 108.6%, making it an economical, instrument-free, and highly adaptable detection strategy.

##### Fluorescence-Based Aptamer Biosensor

Fluorescence technology is one of the most commonly used biosensing methods, characterized by high sensitivity, strong selectivity, and real-time detection capability. By integrating fluorescence technology into sensing probes, the process of biorecognition is converted into fluorescence signal enhancement or attenuation. Combining fluorescence technology with aptamers, researchers have developed various aptamer-based fluorescence sensing strategies for the detection of CEA. Based on the mode of fluorescence signal variation, fluorescent biosensors can be mainly categorized into two types: “Switch-on/off” sensors, in which the presence of the target molecule (CEA) leads to fluorescence signal quenching or increase, and FRET (Fluorescence Resonance Energy Transfer) sensors, which rely on energy transfer between a donor and an acceptor, where target binding influences the FRET effect, resulting in fluorescence signal changes. The variation in fluorescence signals directly reflects the interaction between the target and the aptamer, enabling the quantitative detection of CEA.

##### Switch-On/Off Fluorescence Biosensor

In fluorescence biosensing, the switch-on/off mechanism refers to a significant change in fluorescence signals during target detection, typically manifested as fluorescence enhancement (switch-on) or quenching (switch-off). The switch-off mechanism usually occurs due to interactions between the fluorescent probe and a quencher (such as nanomaterials or metal ions), leading to fluorescence suppression. Conversely, the switch-on mechanism is observed when the addition of the target analyte disrupts this quenching effect, resulting in fluorescence recovery or enhancement. Miao et al. developed a label-free fluorescence sensing strategy based on carbon dots (CDs) for the highly sensitive detection of CEA [192]. In this study, CDs were employed as fluorescent probes, while CEA aptamers (“b1-18 (5′ primer)”, see Table 2) were adsorbed onto the CDs’ surface via π-π stacking interactions, leading to fluorescence quenching (switch-off mechanism). Upon the introduction of CEA, its binding affinity to the aptamer was stronger than the interaction between the aptamer and CDs, causing the aptamer to detach from the CDs’ surface, thereby restoring the fluorescence signal (switch-on mechanism). This method exhibited a good linear relationship in the CEA concentration range of 1 ng/mL to 500 μg/mL, with a detection limit as low as 0.3 ng/mL, demonstrating high sensitivity, excellent selectivity, and reusability. Furthermore, this strategy was successfully applied to the detection of CEA in human serum samples, confirming its potential application in biomedical diagnostics.

In recent years, surface-enhanced fluorescence (SEF) has gained significant attention in the field of bioanalysis, particularly for tumor marker detection. Yang et al. developed an innovative SEF strategy utilizing AuNPs to enhance the fluorescence signal of silver nanoclusters (AgNCs), enabling highly sensitive detection of CEA, as shown in Figure 2B [193]. In this approach, AgNCs serve as fluorescent probes, while AuNPs, modified with DNA, act as fluorescence enhancers. CEA aptamers (“b1-18 (5′ primer)”, see Table 2) link AuNPs and AgNCs, forming an SEF system that amplifies the fluorescence signal. Upon the presence of CEA, its stronger binding affinity to the aptamer displaces the aptamer from AuNPs, disrupting the SEF effect and leading to a decrease in fluorescence intensity, thereby enabling quantitative detection of CEA. This method exhibits a linear response in the CEA concentration range of 0.01–1 ng/mL, with a detection limit of 3 pg/mL, demonstrating high sensitivity, excellent selectivity, and strong anti-interference performance.

##### FRET Biosensor

FRET is an optical phenomenon based on non-radiative energy transfer, in which an excited energy donor transfers energy to an energy acceptor through dipole-dipole interactions. The prerequisite is that the distance between the two is typically within 1–10 nm, and there must be a certain overlap between the emission spectrum of the donor and the absorption spectrum of the acceptor. Due to its high sensitivity, high resolution, and real-time detection capability, FRET technology has been widely applied in biosensing, protein interaction studies, nucleic acid detection, and clinical diagnostics.

Xu et al. proposed an innovative “switch-on” FRET aptasensor for the simultaneous detection of multiple tumor markers (AFP and CEA) [194]. This sensor employs molybdenum disulfide (MoS_2_) nanosheets as the fluorescence acceptor and utilizes green-emitting (510 nm) and red-emitting (650 nm) gold nanoclusters (AuNCs) functionalized with AFP and CEA aptamers (“b1-18 (5′ primer)”, see Table 2), respectively, as energy donors. In the absence of target analytes, MoS_2_ effectively quenches the fluorescence of Au NCs through static quenching effects. However, in the presence of AFP or CEA, the aptamers preferentially bind to their respective targets, leading to their dissociation from the MoS_2_ surface and subsequent fluorescence recovery of AuNCs.

In recent years, upconversion fluorescence resonance energy transfer (UC-FRET) technology has demonstrated excellent sensitivity and specificity in the field of biosensing, leading to its widespread application. Researchers have continuously optimized the UC-FRET system and explored different fluorescence quenchers to enhance detection performance. Wu et al. proposed a UC-FRET-based aptasensor for carcinoembryonic antigen (CEA) detection (the name of aptamer is b1-18 (5′ primer), see Table 2), utilizing NaYF_4_:Yb,Er upconversion phosphors (UCPs) as the energy donor and carbon nanoparticles (CNPs) as the fluorescence quencher [195]. Through π-π stacking interactions, the CEA aptamer adsorbed onto the CNP surface, enabling highly sensitive detection of CEA. In the presence of CEA, the aptamer preferentially binds to CEA, leading to its dissociation from the CNP surface, thereby blocking energy transfer and restoring the fluorescence signal. This method exhibited a good linearity in the range of 0.1–40 ng/mL and was successfully applied to human serum samples, laying the foundation for the application of the UC-FRET system in CEA detection. Wang et al. further optimized the UC-FRET system by employing graphene oxide (GO) as the fluorescence quencher [196]. The π-π stacking interactions between GO and the CEA aptamer (the sequence is shown in Table 2 and named b1-18 (5′ primer)) facilitated efficient energy transfer between UCPs and GO. Upon the introduction of CEA, the aptamer underwent a conformational change and dissociated from the GO surface, inhibiting the FRET effect and restoring the fluorescence signal of UCPs. This method demonstrated a good linear detection range of 0.03–6 ng/mL, with a detection limit as low as 7.9 pg/mL, and exhibited excellent detection consistency in human serum samples, showing a high correlation with commercial chemiluminescence assays (R^2^ = 0.9982). Li et al. explored palladium nanoparticles (PdNPs) as a novel fluorescence quencher and established a highly efficient UC-FRET system based on the strong coordination interaction between PdNPs and the CEA aptamer (named b1-18 (5′ primer), see Table 2) [197]. This method exhibited good linearity in the ranges of 2–100 pg/mL (buffer solution) and 4–100 pg/mL (human serum), with detection limits as low as 0.8 pg/mL and 1.7 pg/mL, respectively, further enhancing the sensitivity of CEA detection.

##### Chemiluminescence-Based Aptamer Biosensor

Chemiluminescence (CL) is a phenomenon in which photons are directly emitted during a chemical reaction, without the need for an external light source. Compared to fluorescence, CL offers advantages such as low background interference, high signal-to-noise ratio, and high sensitivity, making it widely applicable in biomedical detection, environmental monitoring, and chemical analysis.

Zhou et al. utilized chemiluminescence in combination with aptamer technology to develop a highly sensitive detection method for carcinoembryonic antigen (CEA) using capillary electrophoresis-chemiluminescence (CE-CL) [198]. The sequence of the used CEA aptamer is b1-18 (5′ primer), as shown in Table 2. They constructed an HRP-DNA aptamer conjugate, which specifically binds to CEA, forming an HRP–Apt–CEA complex. In the presence of graphene oxide (GO), chemiluminescence resonance energy transfer (CRET) is utilized to quench background signals, while the specific binding event restores the chemiluminescence signal. This method demonstrated a good linear response in the range of 0.0654–6.54 ng/mL, with a detection limit as low as 4.8 pg/mL, and was successfully applied to patient serum analysis. In further studies, Zhou et al. optimized the capillary electrophoresis conditions by adjusting the pH of the electrophoretic buffer and the separation voltage, which significantly improved the separation efficiency of the HRP-DNA_A-B_-QD probe and the CEA/HRP-DNA_A-B_-QD complex [199]. These optimizations reduced overlapping electrophoresis peaks and nonspecific interference, enhancing the detection sensitivity and accuracy of the assay. The method demonstrated a detection limit of 8 pg/mL and was successfully applied to the quantification of CEA in patient serum samples, showcasing its clinical reliability and potential for biomolecule analysis.

In recent years, multiple studies on chemiluminescence aptamer biosensors (CLABs) have integrated nanomaterials, magnetic separation techniques, and signal amplification strategies to enhance the performance of CEA detection and explore the possibility of simultaneous multi-biomarker detection. Man et al. developed a time-resolved chemiluminescence enzyme-linked aptamer assay (TR-CLEIA) based on a dual-enzyme catalytic chemiluminescence system utilizing HRP-aptamer and ALP-aptamer, enabling the simultaneous detection of CEA and VEGF, the aptamer of CEA derived from b1-18 (5′ primer), see Table 2 [200]. This method demonstrated a linear range of 0.5–160 ng/mL for CEA detection with an LOD of 0.1 ng/mL, and was successfully applied to human serum samples, yielding results consistent with commercial ELISA kits. Han et al. designed a CLAB based on metal-organic frameworks (MOFs), where MIL-88B(Fe) loaded with hemin (Hemin@MIL-88B(Fe)) served as a catalyst to significantly enhance the luminol-H_2_O_2_ chemiluminescence signal [201]. This method achieved an LOD of 1.5 × 10^−3^ ng/mL with a linear detection range of 0.01–100 ng/mL, and successfully quantified CEA in serum samples. Shu et al. developed two distinct strategies for ultra-sensitive CEA detection, both employing dual-aptamer sandwich structures but utilizing different nanoparticles for signal amplification [202]. In the first method, Ni NPs catalyzed the luminol-H_2_O_2_ chemiluminescence reaction, achieving a detection limit of 0.092 pg/mL. In the second method, Au NPs were incorporated via a streptavidin-biotin system, where AuNPs were oxidized to Au^3+^ to further enhance the luminol chemiluminescence signal, resulting in a detection limit of 0.122 pg/mL. These two approaches demonstrated excellent sensitivity and specificity, enabling precise CEA detection even at extremely low concentrations in serum samples. Sun et al. [203] developed a CLAB based on dual-aptamer functionalized magnetic silicon composites, enabling the simultaneous detection of CEA and AFP (the sequence of CEA aptamer is shown in Table 2, named b1-18 (5′ primer)). Their design utilized SiO_2_@Fe_3_O_4_ loaded with AuNPs, along with G-quadruplex DNAzyme (G-DNAzyme) to catalyze the luminol-H_2_O_2_ reaction, thereby enhancing the chemiluminescence signal. This method achieved an LOD of 3.2 × 10^−5^ ng/mL for CEA and 6.7 × 10^−5^ ng/mL for AFP, with a high recovery rate (98.0–106.0%) in real serum sample tests as shown in Figure 2C.

##### Electrochemiluminescence-Based Aptamer Biosensor

Electrochemiluminescence (ECL) is an analytical technique that combines electrochemical reactions with chemiluminescence and is widely used in highly sensitive biosensing applications. The fundamental principle of ECL involves applying a potential to an electrode to induce redox reactions of luminophores, which subsequently interact with co-reactants to form excited-state species. When these excited species return to their ground state, photons are emitted, generating a detectable electrochemically induced luminescence signal. In recent years, ECL technology has achieved significant enhancements in signal amplification and detection sensitivity through nanomaterial modifications and surface enhancement effects. Wang et al. developed a surface-enhanced electrochemiluminescence (SEECL) biosensor utilizing Ru(bpy)_3_^2+^@SiO_2_ nanoparticles as luminophores [204]. The incorporation of gold nanoparticles (AuNPs) exploited localized surface plasmon resonance (LSPR) effects to enhance the emission signal, leading to an increased electron excitation rate and improved photon emission efficiency. As a result, a 30-fold signal amplification was achieved, with a detection limit as low as 1.52 × 10^−6^ ng/mL.

Researchers have further explored multiple signal amplification strategies to enhance detection sensitivity. Zhang et al. developed a novel patchy gold-modified Fe_3_O_4_ (PG-Fe_3_O_4_) nanosphere catalyst and applied it to a paper-based bipolar electrode-electrochemiluminescence (pBPE-ECL) aptasensor for carcinoembryonic antigen (CEA) detection [205]. PG-Fe_3_O_4_ was synthesized via an adsorption-reduction method, forming a Janus structure with high catalytic activity. This structure maintained the excellent magnetic properties of Fe_3_O_4_ while significantly improving its catalytic efficiency for the electrochemical reduction of hydrogen peroxide (H_2_O_2_), with a catalytic rate constant of 3.13 × 10^5^ M^−1^s^−1^. Studies have shown that the patchy gold component enables efficient and specific CEA recognition by immobilizing thiol-modified aptamers via Au-S bonding (the CEA aptamer is shown in Table 2). Additionally, the research team electrodeposited gold nanodendrites at the cathode of the pBPE-ECL sensing platform, further enhancing aptamer immobilization density and detection sensitivity. The aptasensor exhibited a linear response range from 0.1 pg/mL to 15 ng/mL, with a detection limit as low as 0.03 pg/mL.

With the continuous advancement of ECL signal enhancement strategies based on nanomaterials, Zhao et al. further developed an ultrasensitive CEA detection method by integrating enzyme-assisted multiple amplification with a DNA walker mechanism [206]. This sensor utilized CdSe quantum dots (QDs) as signal probes and employed Pb^2+^-mediated DNAzyme cleavage, successfully establishing a “signal-off” detection mode with an exceptionally low detection limit of 0.21 fg/mL. The combination of QDs, highly efficient DNA walkers, and enzyme-assisted amplification significantly enhanced signal amplification and detection specificity. These advancements demonstrate the potential of integrating nanomaterials, DNA nanomachines, and electrochemiluminescence technology for highly sensitive biomarker detection, paving the way for early cancer diagnosis and clinical applications. The sequence of the CEA aptamer used in this article is shown in Table 2, named b1-18 (5′ primer).

##### Surface Plasmon Resonance-Based Aptamer Biosensor

Surface plasmon resonance (SPR) is an optical phenomenon in which polarized light interacts with a metal-dielectric interface, exciting surface plasmon waves. The resonance condition depends on changes in the refractive index of the medium near the sensor surface. As a result, SPR enables highly sensitive, label-free, and real-time detection of biomolecular interactions. This technique has been widely applied in the detection of disease biomarkers. In recent years, the integration of aptamers as recognition elements in SPR biosensors has further improved detection specificity and sensitivity, making it a research hotspot for CEA detection.

Guo et al. fabricated an SPR aptasensor based on a zirconium metal-organic framework (Zr-MOF, UiO-66) embedded with silver nanoclusters (AgNCs) [207]. In this study, a CEA-specific aptamer (the sequence of aptamer derived from b1-18 (5′ primer), as shown in Table 2) was employed as a template to synthesize AgNCs@Apt@UiO-66 nanocomposite, which exhibited excellent biocompatibility, electrochemical activity, and bioaffinity. Moreover, the material formed a two-dimensional nanocomposite, enhancing signal response. The sensor was characterized using electrochemical impedance spectroscopy (EIS) and SPR, achieving electrochemical detection limits of 4.93 pg/mL (DPV) and 8.88 pg/mL (EIS), while the SPR detection limit was 0.3 ng/mL. This dual-functional biosensor exhibited high selectivity, good reproducibility, and stability, successfully detecting CEA in real serum samples. By integrating electrochemical and SPR techniques, this study provided a more comprehensive approach for CEA detection.

Erkal-Aytemur et al. developed an aptamer-based sensor using surface plasmon resonance-enhanced total internal reflection ellipsometry (SPRe-TIRE) for highly sensitive CEA detection [208]. The researchers deposited a gold nanofilm on an SF10 glass substrate and functionalized the surface with CEA-specific aptamers via electrochemical diazonium salt reduction and gold nanoparticle (AuNP) modification; the sequence is shown in Table 2, named b1-18 (5′ primer). This platform combined total internal reflection ellipsometry (TIRE) with SPR, significantly enhancing detection sensitivity. The sensor exhibited excellent performance over a broad linear range of 0.01–500 ng/mL, achieving an ultra-low detection limit of 0.1 pg/mL. Additionally, it demonstrated high selectivity with minimal interference from other biomarkers such as AFP, CA125, and VEGF-165. The successful application of this sensor in human serum samples highlights its strong potential for clinical diagnostics.

#### 3.2.2. Electrochemical-Based Aptamer Biosensor

In the field of biosensors, electrochemical sensors are an important detection technology alongside optical sensors. Compared to optical sensors, electrochemical sensors have gained broader applications in biomarker detection due to their high sensitivity, low cost, portability, and rapid response. Particularly in point-of-care testing (POCT), electrochemical sensors enable fast and cost-effective detection with small sample volumes and without the need for expensive instrumentation, making them highly suitable for clinical diagnostics and on-site testing.

In electrochemical biosensors, aptamers serve as highly specific molecular recognition elements and have been widely employed in biomarker detection due to their high stability and ease of chemical modification. Based on the strategy used for aptamer immobilization on the electrode surface, electrochemical aptasensors can be categorized into free aptamers in solution, direct immobilization, nanomaterial-assisted immobilization, and DNA-based nanostructure immobilization. This review will explore these four immobilization strategies and their applications in the detection of CEA.

##### Free Aptamers in Solution

In this strategy, aptamers remain free in solution. The free aptamers undergo specific binding with their target analytes, which subsequently triggers a series of signal amplification processes, such as toehold-mediated strand displacement reactions, exonuclease-assisted target recycling, or hybridization chain reactions (HCR). These processes enhance the generation and amplification of measurable electrochemical signals, enabling highly sensitive and selective detection of target molecules.

Zhang et al. developed an electrochemical aptasensor based on toehold-aided DNA recycling, where probe four was pre-immobilized on the electrode surface [209]. Upon binding with CEA, the aptamer (b1-18 (5′ primer)) released probe one, which triggered DNA strand displacement reactions. This process facilitated the hybridization of more methylene blue (MB)-labeled probe five onto the electrode surface, amplifying the detection signal. The sensor exhibited a linear range of 0.1–50 ng/mL and an LOD of 20 pg/mL, demonstrating successful application in biological sample analysis. Niu et al. developed a CEA aptasensor based on a dual signal amplification strategy utilizing Exonuclease III (Exo III) and HCR. The used CEA aptamer is derived from b1-18 (5′ primer) as shown in Table 2 [210]. The sensor exhibited a detection range of 10 pg/mL–100 ng/mL and an LOD as low as 0.84 pg/mL. Moreover, it demonstrated high selectivity and excellent recovery rates (96.3–101.0%) in serum sample analysis.

##### Direct Immobilization

In the direct immobilization strategy, aptamers are typically covalently bound to the electrode surface. Upon specific binding to the target, the electrochemical signal at the electrode interface undergoes a change, enabling the detection of the biomarker.

Wang et al. proposed an electrochemical aptasensor for the detection of CEA as shown in Figure 2D, the CEA aptamer is b1-18 (5′ primer) [211]. A thiol-modified DNA aptamer was immobilized on a gold electrode to capture CEA. Subsequently, concanavalin A (ConA) was bound to CEA via sugar-lectin interactions, forming a sandwich structure. Signal amplification was achieved through a horseradish peroxidase (HRP)-catalyzed chemical reaction. The sensor employed differential pulse voltammetry (DPV) for detection, exhibiting a linear response in the range of 5–40 ng/mL with an LOD of 3.4 ng/mL, which is lower than the clinical threshold of CEA in cancer patients’ serum (~10 ng/mL). Furthermore, the sensor demonstrated high detection accuracy in human serum samples, with results consistent with those obtained using a commercial ELISA kit, showing a relative error of less than 13%. While the authors describe the sensor as “label-free,” the use of HRP and ConA for signal generation indicates that it is not strictly label-free but rather avoids the direct use of antibodies for detection.

Yunussova et al. immobilized a hexaethylene glycol-modified single-stranded DNA (18-HEG-ssDNA) aptamer on a gold (Au)-based interdigitated electrode (IDE) to develop a label-free electrochemical impedance spectroscopy (EIS)-based aptasensor. The used CEA aptamer is CEA aptamer (6), as shown in Table 2 [212]. This sensor exhibited high sensitivity and specificity, achieving an LOD of 2.4 pg/mL for CEA in phosphate-buffered saline (PBS, pH 7.6) and 3.8 pg/mL in human serum samples. The sensor demonstrated a short detection time, requiring only 20 min of incubation and less than 3 min for EIS measurement, highlighting its potential for rapid cancer screening. This study indicates that the direct immobilization of aptamers enables highly sensitive CEA detection, and the label-free EIS sensor maintains robust detection performance in complex biological samples such as serum. Thus, it provides a rapid, cost-effective, and user-friendly solution for clinical early-stage cancer detection.

##### Nanomaterial-Assisted Immobilization

In the Nanomaterial-Assisted Immobilization strategy, nanomaterials are employed to enhance aptamer immobilization efficiency on the electrode surface while improving electron transfer capability and detection sensitivity.

Xu et al. developed a sandwich-type aptasensor utilizing hemin-functionalized reduced graphene oxide-gold nanoparticles (Hemin-rGO-AuNPs) and organic-inorganic hybrid nanoflowers (HRP-Cu_3_(PO_4_)_2_ HNF) [213]. Apt1 was immobilized on Hemin-rGO-AuNPs to capture CEA (the aptamer is b1-18 (5′ primer) as shown in Table 2), while Apt2 was conjugated with HRP-Cu_3_(PO_4_)_2_ HNF for further CEA recognition, catalyzing the reaction between 4-chloro-1-naphthol(4-CN) and H_2_O_2_ to amplify the signal. This sensor exhibited a linear detection range of 100 fg/mL to 100 ng/mL, with an LOD as low as 29 fg/mL, demonstrating excellent performance in serum sample analysis. Bahri et al. proposed an immunosensor based on AuNPs@CuMOF combined with a three-dimensional tetrahedral DNA (3D TDNA) structure [214]. The 3D TDNA framework provided a rigid structure to stabilize aptamer conformation, enhancing target binding efficiency. The aptamer is b1-18 (5′ primer) as shown in Table 2. This sensor achieved CEA detection within the range of 0.1 pg/mL to 200 ng/mL, with an LOD of 0.25 pg/mL, and demonstrated high accuracy in serum samples, yielding results comparable to those from a commercial ELISA kit (relative error: 2.26–6.25%). Wang et al. developed a label-free electrochemical biosensor based on gold nanoparticles (AuNPs) modified with polypyrrole-polydopamine (PPy-PDA) composites and polycaprolactone (Ng-PCL) [215]. PDA exhibited excellent self-polymerization properties, forming a functionalized coating on the electrode surface with abundant reactive functional groups, thereby enhancing AuNP loading and facilitating covalent aptamer immobilization. One of the aptamers used is shown in Table 2, named b1-18 (5′ primer). Electrochemical impedance spectroscopy (EIS) was employed as the detection technique, converting the biological recognition events into measurable impedance signals. This sensor achieved a linear detection range from 1 pg/mL to 100 ng/mL, with an LOD as low as 0.234 pg/mL, and demonstrated high stability and accuracy in serum sample analysis.

##### DNA Nanostructure-Based Immobilization

Due to their high programmability, biocompatibility, and stability, DNA nanostructures have been widely applied in the immobilization strategies of electrochemical biosensors. In recent years, researchers have utilized DNA nanostructures to construct aptamer-based sensors to enhance detection sensitivity, stability, and specificity. By integrating different signal amplification strategies, these approaches have achieved ultra-low detection limits and broad linear detection ranges [209]. Zhai et al. developed a biosensor based on a dendrimer-like DNA nanoassembly and G-quadruplex DNAzyme [216]. This sensor achieved a detection range of 2–45 ng/mL and an LOD of 0.24 ng/mL. In serum sample analysis, it demonstrated high consistency with ELISA kits, with an agreement rate of 80.7–111%, and retained 96.5% of its signal intensity after 31 days of storage, indicating excellent stability. The used aptamer is also derived from b1-18 (5′ primer). Zhang et al. constructed an enzyme-free electrochemical aptasensor based on tetrahedral DNA nanostructures (TDN) and catalytic hairpin assembly (CHA); the aptamer used is also derived from b1-18 (5′ primer) [217]. This sensor exhibited an ultra-wide detection range of 1 pg/mL–30,000 pg/mL and an LOD as low as 0.04567 pg/mL, demonstrating high sensitivity and specificity in serum sample detection. The DNA nanostructure-based immobilization strategy not only enhances the performance of electrochemical aptamer sensors but also provides novel insights for the advancement of biosensing technologies. Future work can focus on further optimizing nanostructure designs and integrating novel signal amplification mechanisms to develop more efficient, cost-effective, and portable biosensors, thereby offering robust support for precision medicine and early disease diagnosis [210].

### 3.3. MUC1 Detection via Aptamer-Based Biosensors

#### 3.3.1. Aptamer-Based Optical Biosensor

Aptamer-based optical biosensors utilizing colorimetric and fluorescent responses have demonstrated significant potential for detecting MUC1, a critical cancer biomarker, by integrating the specific recognition capabilities of aptamers with visually or spectroscopically detectable signals.

##### Colorimetric-Based Aptamer Biosensor

Li et al. developed a dual-mode aptamer biosensor based on SERS and colorimetry for the detection of the cancer biomarker MUC1 [218]. This sensor utilizes magnetic nanoparticles (MB@SiO_2_@aptamer) as the capture substrate, where MUC1-specific aptamers (“MUC1 S1.3/S2.2”, see Table 2) capture the target molecule. Simultaneously, 5,5′-dithiobis-(2-nitrobenzoic acid) modified gold-silver core-shell nanoparticles (Au@DTNB@Ag-cDNA) serve as SERS probes, generating SERS signals and colorimetric changes through DNA hybridization with the aptamer. By combining the high sensitivity of SERS with the rapid screening capability of colorimetry, the sensor achieves an LOD of 0.1 U/mL and a linear range of 0.1–500 U/mL. This study demonstrates the potential of the sensor in early cancer diagnosis. Ye et al. advanced the field by developing a simplified yet effective colorimetric aptasensor for MUC1 detection. Their sensor employs magnetic beads (MB) and AuNPs to establish a competitive binding system [219]. In the presence of MUC1, the target molecule binds to the aptamer, resulting in the release of AuNPs@Apt-HRP from the MB. This release triggers a colorimetric reaction with TMB, enabling both visual and quantitative detection of MUC1. The sensor exhibits a linear range of 75–500 μg/mL and an LOD of 41.95 μg/mL, offering a cost-effective and rapid alternative for MUC1 detection in clinical settings.

##### Fluorescence-Based Aptamer Biosensor

**Figure 3:**
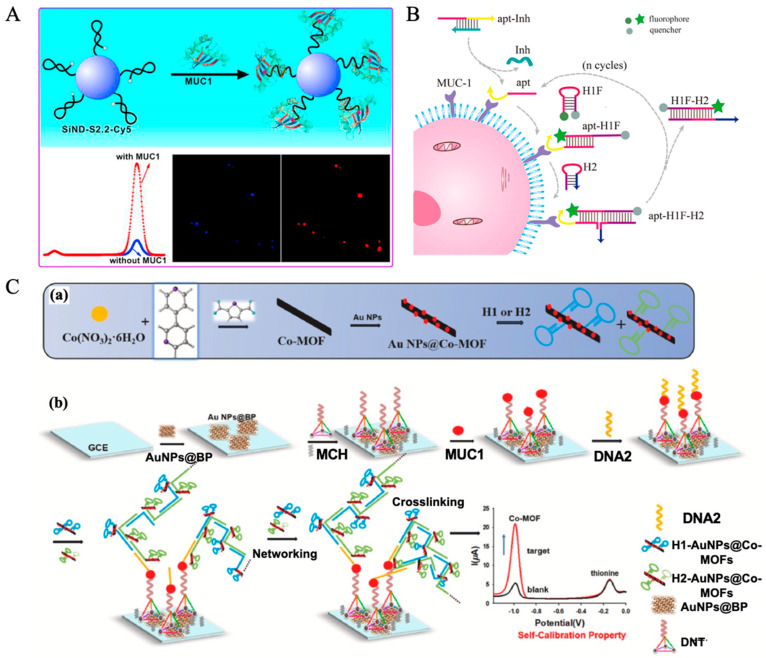
Aptamer-based biosensors for MUC1 detection, emphasizing strategies in fluorescent and electrochemical biosensor technologies. (**A**). Schematic presentation of the SiND-S2.2–Cy5 aptasensor for detection of MUC1 [220]. Copyright © 2018 Elsevier B.V. All rights reserved. (**B**). Schematic illustration of cancer cell detection based on the bifunctional aptamer and CHA [222]. Copyright © 2018 Elsevier B.V. All rights reserved. (**C**): (**a**)Workflow of the synthesis of Co-MOFs, (**b**) Fabrication of the ratio metric aptasensor [224]. Copyright © 2022 Elsevier B.V. All rights reserved.

Zhang et al. developed a dual-color fluorescence aptasensor based on silicon nanodots (SiND) for the detection of the tumor biomarker MUC1 and cancer cell imaging, as shown in Figure 3A [220]. This sensor was constructed by covalently linking Cy5-labeled aptamer S2.2 to SiND, forming the SiND-S2.2-Cy5 composite. In the absence of MUC1, the aptamer (“MUC1 S1.3/S2.2”, see Table 2) maintains a hairpin structure, quenching Cy5 fluorescence; however, in the presence of MUC1, the aptamer undergoes a conformational change, restoring Cy5 fluorescence. This method achieved an LOD of 1.52 nM, exhibiting high sensitivity and selectivity, and was successfully applied for MUC1 detection in human serum samples and imaging of MCF-7 cancer cells. Wang et al. developed a fluorescence detection method based on gold nanoparticles (AuNPs) and carbon dots (CDs), which simplifies the detection process and enhances sensitivity [221]. This approach utilizes the inner filter effect (IFE) of AuNPs on CDs fluorescence, modulated by the specific binding of MUC1 to an aptamer (“MUC1 S1.3/S2.2”, see Table 2), to regulate the aggregation state of AuNPs and achieve a “signal-on” fluorescence response. The method exhibits an LOD of 5.3 ng/mL, along with high selectivity and sensitivity, and has been successfully applied for MUC1 detection in human serum samples.

Aptamer-based biosensors are capable of detecting not only free proteins but also MUC1 proteins on cell membranes and exosome surfaces, offering new possibilities for cancer-related research.

Liu et al. developed a homogeneous fluorescence biosensor based on a bifunctional aptamer and catalytic hairpin assembly (CHA) for the direct detection of MUC1 on cell membranes, as shown in Figure 3B [222]. This sensor employs the bifunctional aptamer (derived from “MUC1 S1.3/S2.2”, see Table 2) to recognize target cancer cells and triggers the CHA reaction for exponential signal amplification, achieving an LOD of 10 cells/mL with high selectivity and sensitivity, and has been successfully applied to cancer cell detection in clinical samples. In contrast, Zhang et al. designed an “on-off” fluorescence aptasensor targeting MUC1 on exosome membranes [223]. This sensor utilizes the specific binding of the MUC1 aptamer to exosomal surface MUC1, inducing a conformational change that separates the fluorophore from the quencher, thereby generating a fluorescence signal. With a LOD of 4.2 × 10^4^ particles/μL, it exhibits high sensitivity and specificity, and has been effectively used for exosome detection in serum samples from breast cancer patients.

#### 3.3.2. Electrochemical-Based Aptamer Biosensor

In the detection of MUC1, aptamer-based electrochemical biosensors exhibit significant advantages due to their high sensitivity and rapid response. These sensors detect the target through changes in electrochemical signals, such as current, impedance, or potential. Furthermore, the modification of electrode surfaces with nanomaterials (e.g., gold nanoparticles or carbon nanotubes) enhances signal amplification and detection sensitivity, rendering them highly promising for MUC1 analysis in complex biological samples, such as serum. The following sections will detail specific applications of electrochemical aptamer-based sensors in MUC1 detection.

##### Voltammetric-Based Electrochemical Aptamer Biosensors

Voltammetric-based aptamer biosensors detect MUC-1 by measuring changes in electrical current resulting from the specific binding of aptamers to the target. These sensors, often employing voltammetric techniques such as cyclic voltammetry (CV) or differential pulse voltammetry (DPV), offer rapid response times and high sensitivity, making them suitable for real-time monitoring in complex samples. Xie et al. developed a ratiometric electrochemical aptasensor for ultrasensitive detection of MUC1, using Co-MOFs as electroactive signal tags and thionine as an internal reference, shown in Figure 3C [224]. The sensor was constructed on a gold nanoparticle-decorated black phosphorus (AuNPs@BP) modified glassy carbon electrode (GCE), with DNA tetrahedral nanostructures (DTNs) for stable aptamer (“MUC1 S1.3/S2.2”, see Table 2) immobilization. In the presence of MUC1, a hybridization chain reaction (HCR) was triggered, bringing Co-MOF-labeled DNA probes close to the electrode surface, generating a cathodic signal at −1.1 V. Thionine provided a stable reference signal at −0.1 V. DPV was used to record both signals, and their ratio enabled self-calibration. The sensor exhibited a linear range of 0.004–400 pM, a detection limit of 1.34 fM, excellent reproducibility (RSD = 2.86%), and high specificity, showing negligible response to non-target proteins such as AFP, CEA, and PSA at 20 pM. In addition, the sensor achieved high recovery (99.8–104.5%) in human serum samples spiked with known concentrations of MUC1. It was further applied to real serum samples from lung cancer patients, where the detected MUC1 level was approximately 12.46 nM after dilution correction, consistent with results obtained from commercial ELISA kits. These results demonstrate its strong potential for clinical application in early cancer diagnostics.

##### Impedance-Based Electrochemical Aptamer Biosensors

Paimard et al. developed an impedance-based electrochemical aptasensor for MUC1 detection, utilizing core-shell nanofibers (NFs) prepared via electrospinning as the electrode surface modification material, integrated with multi-walled carbon nanotubes (MWCNTs) and AuNPs to enhance electron transfer and signal amplification [225]. The MUC1-specific aptamer (“MUC1 S1.3/S2.2”, see Table 2) was immobilized on the modified electrode surface via amide bonds, and upon MUC1 binding, the aptamer-target interaction induced changes in the charge transfer resistance (R_et_) at the electrode interface. Measured by EIS, the sensor exhibited a response range of 5 to 115 nM and an LOD of 2.7 nM. This sensor demonstrated excellent selectivity and stability in detecting MUC1 in serum samples, with results showing strong consistency with those obtained from the ELISA method.

### 3.4. CA125 Detection via Aptamer-Based Biosensors

#### 3.4.1. Aptamer-Based Optical Biosensor

##### Colorimetric-Based Aptamer Biosensor

Ebrahimi et al. developed a colorimetric aptamer sensor based on salt-induced AuNP aggregation, utilizing aptamer-mediated regulation of AuNP aggregation to achieve label-free detection of CA125 [226], with an aptamer derived from “CA125.1” (see Table 2). This method exhibited a linear detection range of 15–160 U/mL, with an LOD of 14.41 U/mL, and was successfully applied to human serum samples, demonstrating good concordance with ELISA results. Additionally, the sensor exhibited high specificity, effectively distinguishing CA125 even in the presence of potential interferents such as CA15-3, CEA, and PSA.

Tripathi et al. developed a nanozyme-integrated aptamer-based lateral flow assay (ALFA) for the detection of CA125, employing peroxidase-mimicking gold nanoparticles (nanozyme-AuNPs) conjugated with CA125 as the signal probe in a competitive format [227]. In this system, the nanozyme-AuNP–CA125 conjugates compete with free CA125 in the sample for binding to immobilized CA125-specific aptamers on the test line. In the absence of free CA125, more conjugates are captured on the test line, where they catalyze the 3,3′ Diaminobenzidine (DAB) and H_2_O_2_ reaction to generate a strong brown signal. Conversely, in the presence of increasing concentrations of CA125, fewer conjugates are captured, resulting in a weaker colorimetric signal. This inverse relationship enables quantitative detection of CA125 within a linear range of 7.5–200 U/mL, with a detection limit of 5.21 U/mL. The assay was validated using clinical serum samples and showed strong agreement with chemiluminescent ELISA results (R^2^ = 0.925, *p* < 0.0001), highlighting its potential for point-of-care cancer diagnostics.

##### Fluorescence-Based Aptamer Biosensor

Fluorescence-based biosensors enable highly sensitive detection of CA125 by utilizing target-induced fluorescence signal changes. This section describes biosensors based on fluorescence resonance energy transfer (FRET) and upconversion luminescence resonance energy transfer (LRET) mechanisms, in which interactions between the energy donor and acceptor lead to fluorescence quenching or recovery. Hamd-Ghadareh et al. developed an antibody-aptamer fluorescence immunosensor based on the FRET mechanism, utilizing carbon dots (CDs) as the fluorescence donor and PAMAM dendrimer-modified gold nanoparticles (PAMAM-AuNPs) as the fluorescence quencher [228]. In the presence of CA125, the energy transfer between CDs and AuNPs was inhibited, leading to an enhanced fluorescence signal. This sensor exhibited a linear detection range from 1.0 fg/mL to 1.0 ng/mL, with an LOD of 0.5 fg/mL. Moreover, it was successfully applied to human serum samples and ovarian cancer OVCAR-3 cell detection, with a minimum detectable concentration of 400 cells/mL, demonstrating excellent potential for bioimaging and clinical diagnostics.

Zhang et al. further optimized fluorescence detection by developing an upconversion fluorescence aptamer biosensor based on the LRET mechanism, utilizing near-infrared (NIR)-excitable upconversion nanoparticles (UCNPs) as the energy donor and carbon dots (CDs) as the energy acceptor [229], with an aptamer derived from “CA125.1” (see Table 2). π-π stacking interactions between UCNPs and CDs triggered the LRET process, leading to fluorescence quenching, whereas in the presence of CA125, this interaction was disrupted, restoring the fluorescence signal. This sensor exhibited a detection range of 0.01–100 U/mL with an LOD of 9.0 × 10^−3^ U/mL, and was successfully applied to human serum sample detection, demonstrating outstanding sensitivity, selectivity, and resistance to autofluorescence interference.

##### Electrochemiluminescence-Based Aptamer Biosensor

Electrochemiluminescence (ECL) biosensors enable highly sensitive detection by combining electrochemical and luminescence principles, where an applied voltage triggers luminescent reactions for signal generation. Zhang et al. developed a DNA tetrahedra-enhanced ECL aptasensor for CA125 detection, integrating toehold-mediated strand displacement (TMSD) and AuNPs/Ru/ZIF-MOF for signal amplification [230], with an aptamer derived from “CA125.1” (see Table 2). Initially in a “signal-off” state, the sensor recovered the ECL signal upon CA125 binding, enabling high sensitivity. It exhibited a linear range of 0.01–10,000 pg/mL with a LOD of 0.006 pg/mL, excellent selectivity, and stability over seven days. Applied to human serum samples, it achieved 93.0-106.6% recovery, demonstrating its potential for clinical cancer diagnostics.

**Figure 4:**
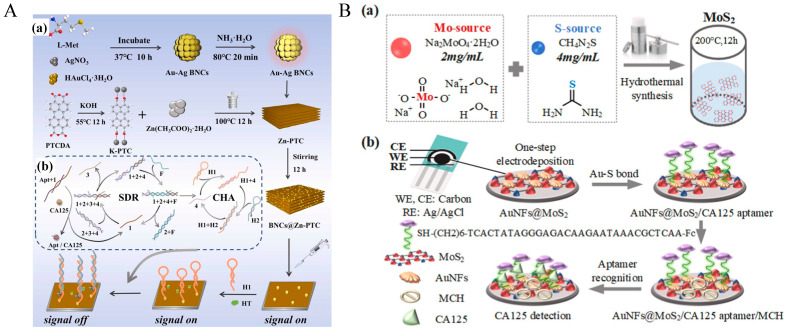
Aptamer-based biosensors for CA125 detection, showcasing both ECL (Electrochemiluminescence)−based and electrochemical-based aptamer biosensing approaches. (**A**): (**a**) The schematic figure of the preparation of luminophores and assembly of the ECL biosensor. (**b**) SDR-CHA dual signal amplification process [231]. Copyright © 2024 Elsevier B.V. All rights are reserved, including those for text and data mining, AI training, and similar technologies. (**B**): Schematic diagrams of the sensing platform. (**a**) Synthesis of MoS2. (**b**) Modification of AuNFs@MoS2/CA125 aptamer/MCH for CA125 detection [235]. Copyright © 2023 Elsevier Inc. All rights reserved.

To enable ultrasensitive clinical detection of CA125 in PC diagnostics, Chen et al. developed an electrochemiluminescence resonance energy transfer (ECL-RET) biosensor for CA125 detection, as shown in Figure 4A [231], with an aptamer derived from “CA125.1” (see Table 2). The introduction of the ECL-RET system advances the development of electrochemiluminescence biosensing by significantly reducing the luminescence potential while improving sensitivity and signal stability. This study introduced a BNCs@Zn-PTC composite material, where Zn-PTC served as the ECL energy donor, and Au-Ag bimetallic nanoclusters (BNCs) acted as the energy acceptor, achieving efficient ECL-RET through spectral overlap, which further lowered the luminescence potential and enhanced the ECL signal. By integrating a strand displacement reaction–catalyzed hairpin assembly (SDR-CHA) dual signal amplification strategy, the sensor exhibited a linear detection range from 1 μU/mL to 10 U/mL, with an ultrasensitive detection limit of 0.24 μU/mL. Furthermore, the biosensor demonstrated excellent recovery rates (100.28–107.24%), along with high specificity, stability, and reproducibility in human serum samples, providing a promising approach for clinical PC detection and respectability assessment.

##### Surface Plasmon Resonance-Based Aptamer Biosensor

Valizadeh Shahbazlou et al. developed a biotinylated aptamer-based SPR biosensor for CA125 detection, using a streptavidin-coated gold chip for aptamer (derived from CA125.1, see Table 2) immobilization [232]. The sensor was optimized for temperature, flow rate, and buffer pH, achieving a linear range of 10–100 U/mL and a LOD of 0.01 U/mL. It demonstrated high precision, accuracy (97.5–105% recovery), and selectivity against interfering biomarkers (CEA, leptin, CA19-9). Successfully applied to human serum samples, it shows promise for clinical cancer screening. Compared to antibody-based methods, this aptasensor offers higher specificity, a broader detection range, and improved sensitivity, making it a valuable tool for real-time CA125 quantification.

#### 3.4.2. Electrochemical-Based Aptamer Biosensor

##### Voltammetric-Based Electrochemical Aptamer Biosensors

In recent years, electrochemical aptamer-based sensors using voltammetry have demonstrated excellent sensitivity and selectivity for CA125 detection. Researchers have employed various nanomaterials to modify electrodes and integrated different signal amplification strategies, such as target-triggered strand displacement amplification (SDA), to achieve effective signal enhancement. Chen et al. developed an electrochemical aptasensor for CA125 by modifying a screen-printed carbon electrode with flower-like gold nanostructures (AuNSs) to increase probe loading and signal efficiency. The system employed target-triggered SDA and square wave voltammetry (SWV) for signal readout. The electrochemical signal was generated by methylene blue (MB), an electroactive molecule that was covalently labeled at the 3′ end of a DNA hairpin probe (Hp2). Upon target binding and SDA activation, MB-labeled Hp2 was brought into close proximity to the electrode surface, producing a measurable current. The sensor achieved a detection limit of 5.0 pg/mL and showed good specificity and recovery in spiked biological samples, with an aptamer derived from CA125.1 (see Table 2) [233]. Zhang et al. [234] developed a label-free electrochemical aptasensor based on a Nickel hexacyanoferrate–polydopamine functionalized graphene (NiHCF/PDA@Gr) composite, in which NiHCF nanocubes functioned as an in situ electroactive signal probe immobilized on the electrode surface. The redox signal originated from the reversible Fe^3+^/Fe^2+^ redox couple in NiHCF, without the need for any externally added or labeled electroactive species. The PDA@Gr matrix enhanced electron transfer and surface area for aptamer (derived from CA125.1, see Table 2) immobilization, enabling a highly sensitive detection of CA125 with a detection limit of 0.076 pg/mL [234]. Additionally, Hu et al. modified the electrode with an AuNFs@MoS_2_ nanocomposite and employed differential pulse voltammetry (DPV) for CA125 detection. In this system, the electrochemical signal was generated by ferrocene (Fc), which was covalently tagged to the 3′ end of the CA125-specific aptamer. Upon target binding, the aptamer underwent a conformational change, bringing Fc closer to the electrode surface and enhancing the redox current. This allowed for quantitative analysis over a wide concentration range from 0.0001 to 500 U/mL, as shown in Figure 4B [235]. These studies indicate that voltammetric methods, combined with nanomaterials and nucleic acid amplification strategies, can significantly enhance the performance of CA125 sensors.

##### Impedance-Based Electrochemical Aptamer Biosensors

Researchers have employed magnetic nanomaterials and metal oxides to modify electrodes and, in combination with aptamer recognition strategies, have achieved highly sensitive detection of CA125 by monitoring charge transfer resistance (R_et_) variations. Yue et al. modified a glassy carbon electrode (GCE) with a Mg_0.5_Cu_0.5_Fe_2_O_4_-Au nanocomposite, which endowed the electrode with magnetic responsiveness. This enabled magnetically induced self-assembly of the nanocomposite on the GCE surface, improving the sensor’s stability and sensitivity due to the material’s high conductivity and biocompatibility, with an aptamer derived from CA125.1 (see Table 2) [236]. This approach enabled a detection range of 5–125 U/mL with a detection limit of 4.4 U/mL.

### 3.5. Protein-Related Multiple Biomarker Detection via Aptamer-Based Biosensor

While numerous aptamer biosensors have been documented targeting each of the listed biomarkers believed to correlate significantly with PC progression, it is not surprising to expect poor standalone diagnostic specificity towards PC, as these are also common biomarkers found in breast, lung, and colorectal cancer [44,237,238,239]. A possible solution would be to develop multiplex assays to simultaneously monitor key biomarkers and create a patient-specific biomolecular profile for accurate diagnosis and personalized treatment [240,241,242,243]. Yet the development of multiplex aptamer biosensors for PC diagnosis has been rather limited, presumably due to the prevalence of immunosensor research over aptamers. Still, a couple of recent examples have proven the efficacy of multi-target aptamer sensing in PC clinical applications.

**Figure 5:**
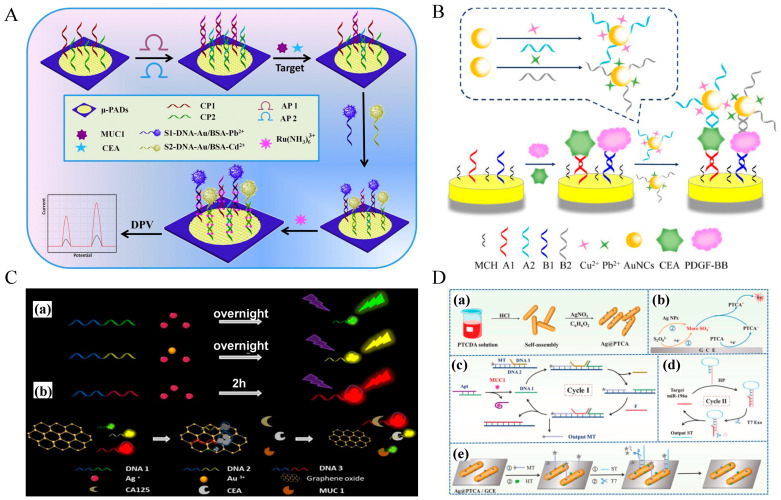
Integrated detection of multiple biomarkers, including combinations of proteins and protein-miRNA complexes. (**A**). Schematic representation of the dual-target electrochemical aptasensor for the detection of carcinoembryonic antigen and mucin−1 based on metal ion electrochemical labels and Ru(NH_3_)_6_^3+^ electronic wires [244]. Copyright ©2017 Elsevier B.V. All rights reserved. (**B**). Scheme of electrochemical aptamer sensor based on metal ion-labeled polyethyleneimine gold nanoparticles for simultaneous detection of multiple disease markers [245]. Copyright © 2021 Elsevier Ltd. All rights reserved. (**C**): (**a**) Schematic illustration of synthesizing DNA−AgNCs and DNA−Ag/AuNCs; (**b**) Schematic demonstration of the mechanism for assaying MUC1, CEA, and CA125 [247]. Copyright © 2018 Elsevier B.V. All rights reserved. (**D**). Schematic of the ECL biosensor for concurrent MUC1 and miRNA-196a detection [249]. (**a**) Synthesis of Ag@PTCA. (**b**) Luminescence signal amplification by AgNPs. (**c**) Stand displacement reaction (SDR) mechanism detecting the MUC1 biomarker. The star conjugated to the MT oligo is the ferrocene (Fc) moiety. (**d**) T7 exonuclease mediated detection of miRNA−196a. (**e**) A signal “on−off−on” ECL biosensor for detecting MUC1 and miRNA−196a. Copyright © 2024 Elsevier B.V. All rights reserved.

Ma et al. developed a multiplex electrochemical aptamer biosensor (E-AB) for the simultaneous detection of serum MUC1 and CEA, as shown in Figure 5A [244]. The sensor utilized protein binding-induced strand displacement and subsequent hybridization with complementary strands, coupled with metal ion-loaded nanospheres to generate distinctive electrochemical reduction currents indicating the presence of each target. The proposed aptamer biosensor demonstrated a wide detection range from 0.01 pM to 100 nM, with a remarkably low LOD of 3.33 fM. The miniaturized paper-based design encourages future point-of-care diagnostic applications as a low-cost and portable device.

Similarly, another E-AB model developed by Zhao et al. is shown in Figure 5B [245] and detects and differentiates CEA and platelet-derived growth factor (PDGF) from human serum. The two markers were quantified by the differential electrochemical redox signals from Cu^2+^ and Pb^2+^ nanoprobes, respectively, using voltametric analysis. Although the assay achieved a low pg/mL LOD with decent specificity, its reliance on a macroelectrode system poses challenges for adoption in both clinical and point-of-care settings.

Zou et al. proposed a dual-detection approach for CEA and CA125 from human serum samples [246]. This study featured specific aptamers coated onto magnetic beads and fluorescence carbon dots, leading to sandwich binding in the presence of target antigens. Upon magnetic separation and DNase I digestion, the two fluorescence carbon dots were released from the binding complex, and the fluorescence was measured. CEA and CA125 concentrations were correlated to the fluorescence intensity of the specific aptamer carbon dots. It showed excellent sensitivity for CEA and CA125 at 0.11 ng/mL and 0.57 U/mL, respectively. Promising diagnostic consistency was observed in differentiating cancer from healthy serum samples compared to a standard clinical immunoassay.

A triplex graphene oxide FRET assay developed by Wang et al. allowed simultaneous detection of MUC1, CEA and CA125 as shown in Figure 5C [247]. Three specific aptamers conjugated to metal nanoclusters recovered strong fluorescence at different wavelengths when displaced from the graphene oxide sheet upon target binding. The proposed method exhibited excellent analytical performance with detection limits of 0.18 ng/mL for MUC1, 3.18 ng/mL for CEA, and 1.26 ng/mL for CA125, demonstrating its potential for clinical diagnostics and multiplexed tumour marker detection.

Li et al. reported a point-of-care lateral flow assay (LFA) combined with SERS for the simultaneous detection of VEGF and osteopontin [248]. Specific aptamers were printed into separate test lines to allow dual antigen detection through sandwich binding with aptamer-decorated cuprous oxide nanocubes (Cu_2_O NCs). In the presence of the target antigens, clustering of the reporters on the test line generates a visible yellow band for qualitative observation by the naked eye and quantitative analysis by SERS. The assay achieves rapid detection within 15 min, with LODs of 0.78 pg/mL for VEGF and 0.86 pg/mL for OPN, which are well below clinically required levels. Additionally, the biosensor demonstrates excellent specificity, reproducibility, uniformity, and stability. It was successfully applied to clinical serum samples, effectively distinguishing cervical cancer patients from healthy individuals.

Apart from the detection of Protein-protein combination, Chen et al. reported a concurrent detection model against serum MUC1 and microRNA-196a to improve the diagnostic accuracy of singleplex MUC1 biosensing for pancreatic cancer, as shown in Figure 5D [249]. The model employed strand displacement reaction (SDR) cascades to detect the presence of both MUC1 and microRNA-196a from human pancreatic adenocarcinoma cells (PANC-1). The study signified the catalytic property of silver nanoparticles and target recycling SDR cascades to amplify the electroluminescence signal. As a result, the biosensor achieved detection limits of 0.63 fg/mL for MUC1 and 4.57 aM for miRNA-196a and successfully differentiated PANC-1 from healthy pancreatic cells and hepatocellular carcinoma cells, highlighting the selectivity benefits from the dual-target detection approach. Besides being sensitive, the assay is better suited to a clinical setting due to its complexity and the need for a specific measuring instrument.

Singleplex aptasensors benefit from facile assay development and straightforward result interpretation. However, it is evident that mono-biomarker tracking leads to poor diagnostic and prognostic performance in PC due to population heterogeneity. As more aptamers are being selected and developed for biosensors for PC diagnosis, a promising approach is the simultaneous tracking of key markers using a panel analysis with target-specific aptamers to capture the complexity and variability of PC progression at different stages across individuals. It has significant potential to improve the accuracy and reliability of PC prediction in the future.

### 3.6. Exosomes as Biomarkers Detection via Aptamer-Based Biosensors

Exosomes are nanoscale vesicles (30–150 nm) that carry proteins, nucleic acids, and metabolites from their parent cells, providing a novel perspective for the detection of pancreatic cancer [250]. In recent years, aptamer-based sensing technology has shown significant advantages in the detection of exosome markers through the combination of molecular recognition and signal amplification strategies. The technological evolution of pancreatic cancer exosome aptasensors has progressed from single-biomarker detection to multi-biomarker collaborative recognition, and from reliance on complex preprocessing to direct analysis of clinical samples, marking a paradigm of iterative innovation.

#### 3.6.1. Single-Biomarker Recognition via Aptamer Biosensor

**Figure 6:**
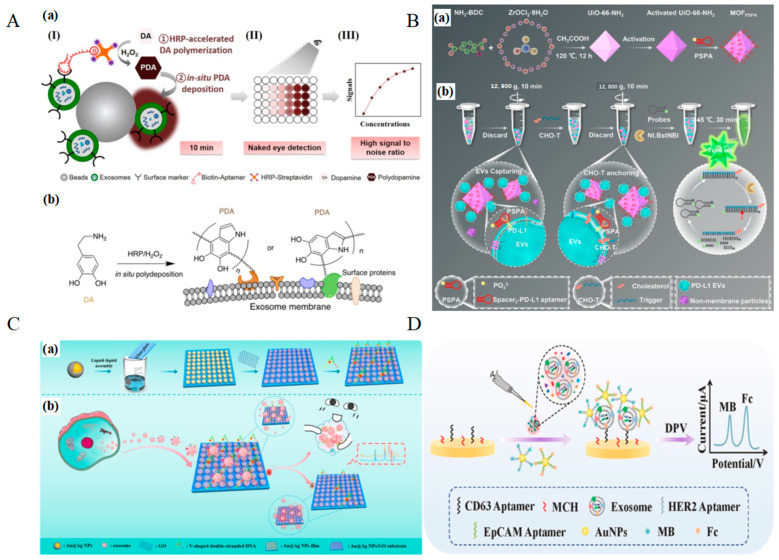
Schematic illustrations of advanced strategies for exosome detection. (**A**). Schematic illustration of the proposed aptasensor with HRP-accelerated dopamine polymerization and deposition for exosome detection: (**a**) Exosome capture via CD63-aptamer/HRP complex, (**I**–**III**) Colorimetric readout enabling visual detection and absorbance quantification; (**b**) HRP-catalyzed DA polymerization into PDA. [252]. Copyright © 2020 Elsevier B.V. All rights reserved. (**B**). Schematic illustration of (**B**): (**a**) the synthesis of MOFPSPA and (**b**) EV–ANCHOR for the isolation and analysis of PD–L1 EVs [253]. Copyright © 2023 Elsevier B.V. All rights reserved. (**C**). Schematic Illustration of the Mechanism of Ratiometric SERS Biosensor for Exosome Detection: (**a**) Construction of Au@Ag NPs/GO SERS substrate; (**b**) Exosome-triggered dissociation of ROX-labeled V-shaped DNA duplex [255]. Copyright © 2023, American Chemical Society. (**D**) Schematic illustration of the one-step multiplex analysis for breast cancer exosomes based on an electrochemical strategy assisted by AuNPs [256]. Copyright © 2023 Elsevier B.V. All rights reserved.

The initial phase of pancreatic cancer exosome detection focused on the identification of single biomarkers. For instance, Shin et al. developed two distinct aptamer-based methods for ALPPL2 detection in pancreatic cancer-derived extracellular vesicles [251]. The first approach utilized a direct aptamer-linked immunosorbent assay (ALISA) with the SQ2 aptamer for exosome capture and streptavidin-HRP signal amplification, achieving a sensitivity of 10 ng/mL (protein concentration). The second method employed a sandwich ALISA format combining CD9 antibody-mediated exosome capture and SQ2 aptamer-based detection, which significantly improved sensitivity to 100 pg/mL. This dual-method validation confirmed ALPPL2 as a robust diagnostic biomarker for pancreatic cancer. Xu et al. introduced a colorimetric aptasensor targeting integrin αvβ6, a potential pancreatic cancer exosomal biomarker, as shown in Figure 6A [252]. By conjugating αvβ6-specific aptamers to horseradish peroxidase (HRP) via biotin-streptavidin binding, their platform enabled direct exosome capture on latex beads and signal amplification through localized polydopamine (PDA) deposition.

However, the reliance on ultracentrifugation for exosome isolation and single-target detection limited its applicability to heterogeneous clinical samples. This technological trajectory was further advanced by Feng et al., who developed the EV-ANCHOR platform using PD-L1 aptamer-functionalized metal-organic frameworks (MOFs) combined with cholesterol-triggered signal amplification, as shown in Figure 6B [253]. While Shin’s ALISA required 70-min ultracentrifugation and Xu’s system achieved direct clinical sample analysis, EV-ANCHOR eliminated centrifugation entirely through Zr-O-P coordination chemistry, enabling PD-L1^+^ exosome isolation by specific aptamer recognition within 10 min via simple microcentrifugation (12,800× *g*). Cholesterol-modified DNA strands were anchored onto the exosome and allowed FAM-BHQ1 probing strand hybridization. Specific endonuclease digestion by Nt.BstNBI cleaved the probes and recovered the FAM fluorescence intensity, directly proportional to the PD-L1 exosome concentration. The cholesterol-mediated signal amplification achieved a sensitivity of 9.4 × 10^4^ particles/μL, surpassing Xu’s PDA-based system (7.7 × 10^3^ particles/mL). While these single-biomarker approaches have laid the foundation for exosome-based diagnostics in pancreatic cancer, their limitations, such as vulnerability to tumor heterogeneity and dependence on laborious ultracentrifugation [254], have prompted the field to expand beyond pancreas-specific targets and explore multi-biomarker systems that can enhance diagnostic accuracy and practicality across diverse cancer types.

#### 3.6.2. Multiple-Biomarker Recognition via Aptamer Biosensor

To overcome the limitations of single-target systems, researchers advanced a dual-aptamer colorimetric aptasensor for detecting pancreatic cancer exosomes. Zhang et al. developed a ratiometric surface-enhanced Raman scattering (SERS) biosensor using V-shaped double-stranded DNA probes targeting epithelial cell adhesion molecule (EpCAM) and human epidermal growth factor receptor-2 (HER_2_), as shown in Figure 6C [255]. This unique bivalent aptamer design employs complementary DNA (cDNA) to bridge EpCAM and HER_2_ aptamers, forming a rigid V-shaped architecture that spatially aligns the two aptamers for synergistic binding to exosome surface proteins. Unlike linear dual-aptamer configurations, the V-shaped structure minimizes steric hindrance and enhances binding avidity, enabling precise recognition of exosomes co-expressing both biomarkers. Although initially designed for breast cancer exosomes, this platform demonstrated remarkable versatility by distinguishing pancreatic cancer patients from healthy individuals in clinical serum analysis (AUC = 0.93). The bivalent aptamer-exosome interaction triggers a competitive displacement mechanism, where the stronger affinity between aptamers and target proteins overcomes the π-π interaction between DNA and graphene oxide, leading to probe release and ratiometric signal changes. This design not only improves specificity by requiring dual-target recognition but also eliminates the need for nucleic acid amplification, as confirmed by the ultralow detection limit (1.5 × 10^2^ particles/mL). Zhang et al. further developed a one-step electrochemical aptasensor utilizing a multi-probe recognition strategy to simultaneously detect HER_2_, EpCAM, and CD63 exosomal markers, as shown in Figure 6D [256]. The research team validated the sensor’s performance in distinguishing HER2-positive and HER_2_-negative breast cancer exosomes and assessed its applicability in complex biological samples. The results demonstrated that the sensor achieved a detection limit of 3.4 × 10^3^ particles/mL, exhibiting high specificity, excellent stability, and ease of operation. While this study focused on breast cancer screening, the proposed method can also be applied to the early detection and prognostic evaluation of pancreatic cancer and other malignancies.

The progression from single- to dual-biomarker detection reflects a broader shift toward precision and clinical practicality in pancreatic cancer diagnostics. While early platforms prioritized biomarker validation, contemporary systems emphasize multiplexed detection, signal robustness, and compatibility with complex biological matrices. Emerging technologies, such as CRISPR-based exosome RNA profiling [257,258] and AI-driven spectral interpretation [259], promise to bridge the gap between laboratory innovation and clinical implementation.

## 4. Conclusions

Pancreatic cancer (PC) is characterized by its aggressive onset and lack of specific early symptoms, leading to late-stage diagnosis and poor overall survival rates. Therefore, the identification of highly sensitive and specific biomarkers is crucial for the early detection of PC. As a non-invasive screening method, serum biomarker detection plays a significant role in the diagnosis of pancreatic cancer diagnosis. This review summarizes well-studied serum protein biomarkers (CA19-9, CEA, mucins, and osteopontin) and explores the potential of emerging biomarkers, including exosomes, circulating tumor cells, microRNAs (miRNAs), circulating tumor DNA (ctDNA), and metabolites in pancreatic cancer detection. The sensitivity and specificity of single-marker detection are limited. To address these limitations, multi-biomarker panels have emerged as a promising strategy. In this review, we highlight CA19-9 as a vital anchor in such panels, playing a crucial role in enhancing diagnostic accuracy and enabling personalized early-stage PC diagnosis.

Additionally, we categorize and summarize the applications of aptamer-based (Aptamer) biosensor technology in the detection of pancreatic cancer serum protein biomarkers and analyze its advantages and future directions. In the field of biosensing technology, aptamer-based biosensors have emerged as a novel molecular recognition tool with high affinity, excellent stability, ease of modification, and low cost, demonstrating significant potential in the detection of cancer biomarkers. Aptamer biosensors can be integrated with various detection techniques, among which optical and electrochemical biosensing technologies are currently the most well-developed. This review summarizes the applications of various optical and electrochemical aptamer-based methods for detecting different PC-related biomarkers. Optical aptamer-based biosensors are primarily based on principles such as colorimetry, fluorescence, chemiluminescence, and surface plasmon resonance (SPR). These platforms generate detectable optical signals through the specific binding between aptamers and target molecules, offering high sensitivity and intuitive visualization, which makes them suitable for high-precision analysis in laboratory settings. In contrast, electrochemical aptamer-based biosensors detect targets by monitoring changes in electrical signals, including current, voltage, or impedance. These systems are characterized by their simplicity, ease of integration, and potential for on-site detection. In recent years, the incorporation of nanomaterials has significantly enhanced the performance of both types of sensors. Particularly in electrochemical sensing platforms, the excellent electrical conductivity and favorable surface functionalization properties of nanomaterials have greatly improved the sensitivity and stability of the sensors. Overall, optical methods are more suitable for high-throughput and image-based detection, whereas electrochemical methods show greater potential in portable and point-of-care testing (POCT) applications. Looking ahead, with the integration of multimodal detection strategies and intelligent analytical techniques, aptamer-based biosensors are expected to play an increasingly important role in the early diagnosis of cancer.

## 5. Future Perspectives

The commercialization of aptamer-mediated diagnostic solutions would be a major step forward for real-world applications. While companies like SOMAlogic or Aptamer Group PLC have developed robust pipelines to identify novel aptamers, there are currently very few aptamer-based in vitro diagnostic (IVD) devices approved by regulatory bodies for diagnostic purposes. Particularly for cancer diagnosis, the AptoDetect™-Lung from Aptamer Sciences, Inc. is the only IVD product approved by the South Korean Ministry of Food and Drug Safety (MFDS) at present. This highlights the key challenges in conducting rigorous large-scale clinical validation and overcoming regulatory hurdles for authorization. However, considering the increasing number of publications reporting the development of novel PC aptasensors and multiple products registered as research use only (RUO) are already available in the market, we should expect the commercialization of aptamer-mediated IVD medical solutions to materialize in the future for early-stage PC diagnosis.

Moving forward, research on pancreatic cancer serum biomarkers will continue to advance, focusing on identifying more specific and sensitive biomarkers, as well as incorporating them into multiple biomarker panels. Multiplex biomarker panels integrating CA19-9 with other biomarkers under genetic stratification offer new directions to be investigated for early-stage PC diagnosis. It is essential to develop aptamers and aptasensors further to detect CA19-9 as an indispensable early-stage PC diagnosis biomarker. Furthermore, combining aptamer nanotechnologies with electrochemical sensing technologies offers a promising direction for a point-of-care device for early-stage PC. Although aptamer-based sensing is a promising direction in sensing technologies, off-target and stability issues in serum could hinder the use of natural aptamers in actual biosensing applications. Off-target issues might be solved by selecting aptamers in conditions that mimic actual biological conditions, with extra counter SELEX based on actual biosensing conditions. Chemical modifications on natural aptamers have the potential to solve the aptamer stability problem in serum, while the investigation of chemically modified aptamers in biosensing has been limited to date. Efforts should be directed toward developing aptamers in actual biological conditions, investigating aptamers with chemical modifications in biosensors, optimizing multiplex biomarker detection technologies, integrating various bioinformatics approaches coupled to AI-driven analysis, and advancing clinical validation. A precise and efficient early screening system for pancreatic cancer will ultimately improve patient prognostic outcomes.

## Figures and Tables

**Table 2:** Aptamers for serum-based biomarkers.

Biomarker	Aptamer Sequence (5′-3′)	Aptamer Name	Aptamer Type	Kd (nM)	Kd Measurement Method	Immobilization	Ref.
CA19-9	GACTGGCCCAGGCCCCCTCCTCCCGCTGCTGCCCGCCCTC	CA19-9 aptamer	DNA	20.05 ± 3.02	Fluorescence assay	Polystyrene plate	[166]
CEA	ATACCAGCTTATTCAATT	b1-18 (5′ primer)	DNA	0.69	Fluorescence Polarization (FP)	Free in solution	[167]
	CCGATCCCACCGACCGCGCCCTGCCTCAGCCCCTCCCCGTG	NR *	DNA	41.49 ± 9.97	Fluorescence	Polystyrene plate	[166]
	GGGGGGTGTATCGTTGACGAGTTGCGCGTGCGTCTCGTG	Apta 3	DNA	60.4 ± 5.7	qPCR	Free in solution	[168]
	GGAGCTACGTTTAGCGAGTCCGACGCTCGGTGCCTCTTC	Apta 5	DNA	37.8 ± 5.8	qPCR	Free in solution	[168]
	ATACCAGCTTATTCAATTATG	P-ATG	DNA	4.62	Biolayer Interferometry (BLI)	Streptavidin-coated biosensor (SSA)	[169]
	GACATACCAGCTTATTCAATT	GAC-P	DNA	3.93	BLI	Streptavidin-coated biosensor (SSA)	[169]
	CCGCTACCCCCCACGCAATCCCG	G3S1.5	DNA	2.46	Isothermal titration calorimetry (ITC)	Free in solution	[170]
	GCCAGCGAGTTTTGACCGTTTTTCTCTCTTTTCCGCCTA	Aptamer (6)	DNA	312.6	ELONA	CEA-coated plate	[171]
MUC1-APDTRPAPG epitope	GCAGTTGATCCTTTGGATACCCTGG	MUC1 S1.3/S2.2	DNA	0.135	Surface Plasmon Resonance (SPR)	Biotinylated peptide on streptavidin chip	[172]
MUC1-5TR-GalNAc (Tn antigen)	GGCTATAGCACATGGGTAAAACGAC	5TRG2	DNA	18.6	SPR	Nickel-NTA chip	[173]
Unglycosylated MUC1-5TR peptide (5 tandem repeats)	GAAGTGAAAATGACAGAACACAACA	5TR1	DNA	21.0	SPR	Nickel-NTA chip	[173]
MUC1-N-acetylgalactosamine (GalNAc) alone	AAGGGATGACAGGATACGCCAAGCT	GalNAc3	DNA	47.3	SPR	96-well polystyrene plate	[173]
MUC1-APDTRPAPG epitope	AACCGCCCAAATCCCTAAGAGTCGGACTGCAACCTATGCTATCGTTGATGTCTGTCCAAGCAACACAGACACACTACACACGCACA	MA3	DNA	38.3	Flow cytometry	Peptide conjugated to magnetic beads	[174]
CA125	AGGCGGGCGGCGTGGCGATGTTACTGCGTGTGTGTTCGTG	CA125-aptamer	DNA	17.41 ± 2.26	Fluorescence	Polystyrene plate	[166]
	ACTAGCTCCGATCTTTCTTATCTAC	CA125 1	DNA	207 ± 109 U/mL (FA), 80 ± 38 U/mL (APCE)	Fluorescence Anisotropy (FA), Affinity Probe Capillary Electrophoresis (APCE)	Free in solution	[175]
	TGCCTTATTACTCTCTCCTGTTAAC	CA125 12	DNA	118 ± 123 U/mL (FA), 131 ± 93 U/mL (APCE)	FA, APCE	Free in solution	[175]
	TAGGGAAGAGAAGGACATATGATTTTAGGGAAGAGAAGGACTTTTATGCCGCCTTGACTAGTACATGACCACTTGA	Apt 2.26	DNA	166	Membrane-based assessment of bound ssDNA	Nitrocellulose membrane (0.2 μm pore size) with immobilized CA125	[176]
	ACCACCACCACGACGCACGAGTACCCCGCG	rCAA-8	DNA	166	BLI	Streptavidin-coated optical sensor	[177]
	AAAAUGCAUGGAGCGAAGGUGUGGGGGAUACCAACCGCGCCGUG	CA125.1	RNA	4.13	SPR	Covalent immobilization on CM5 biochip	[178]
Osteopontin	TGTGTGCGGCACTCCAGTCTGTTACGCCGC	C10K2	DNA	2.5 (30 min), 1.1 (4 h)	Fluorescence assay	Streptavidin-biotin interaction on a gold electrode	[179]
	CGGCCACAGAAUGAAAAACCUCAUCGAUGUUG	OPN-R3	RNA	18.00	NR *	NR *	[180]

* NR, not reported.

## Data Availability

No new data were created or analyzed in this study. Data sharing is not applicable to this article.

## Abbreviations

The following abbreviations are used in this manuscript:

5hmC	5-Hydroxymethylcytosine
AUC	Area Under the Curve
BP	Benign Pancreatic Cystic Neoplasms
BPD	Benign Periampullary Disease
CA125	Cancer Antigen 125
CA19-9	Carbohydrate Antigen 19-9
CEA	Carcinoembryonic Antigen
cfDNA	Cell-Free DNA
CLIA	Clinical Laboratory Improvement Amendments
CLIA	Chemiluminescent Immunoassay
CPA	Serum Carboxypeptidase
CRISPR	Clustered Regularly Interspaced Short Palindromic Repeats
CTCs	Circulating Tumor Cells
ctDNA	Circulating Tumor DNA
dCCA	Distal Cholangiocarcinoma
DPV	Differential Pulse Voltammetry
ECL	Electrochemiluminescence
ECLIA	Electrochemiluminescence Immunoassay
EIS	Electrochemical Impedance Spectroscopy
ELISA	Enzyme-Linked Immunosorbent Assay
EVs	Extracellular Vesicles
FDA	Food and Drug Administration
FRET	Fluorescence Resonance Energy Transfer
GLRX3	Glutaredoxin3
GPC-1	Glypican-1
IL	Interleukin
IPMN	Intraductal Papillary Mucinous Neoplasm
IVD	In Vitro Diagnostic
KRAS	Kirsten Rat Sarcoma Viral Oncogene Homolog
LOD	Limit of Detection
LRG1	Leucine-Rich Alpha-2-Glycoprotein 1
LTB4	Leukotriene B4
MFDS	Ministry of Food and Drug Safety
miRNAs	MicroRNAs
MMP	Matrix Metalloproteinase
MUC	Mucin
NGS	Next-Generation Sequencing
PanIN	Pancreatic Intraepithelial Neoplasia
PB	Pancreatic Benign Disease
PC	Pancreatic Cancer
PDAC	Pancreatic Ductal Adenocarcinoma
PHA-E-positive Cp	Phaseolus vulgaris Erythroagglutinin-Positive Ceruloplasmin
POCT	Point-of-Care Testing
PRKCB	Protein Kinase C Beta Type Gene
qRT-PCR	Quantitative Reverse Transcription and Real-Time Polymerase Chain Reaction
RUO	Research Use Only
sCD40	Soluble CD40
sCD163	Soluble CD163
SDC1	Syndecan-1
SELEX	Systematic Evolution of Ligands by Exponential Enrichment
SN	Sensitivity
SP	Specificity
SPR	Surface Plasmon Resonance
SWV	Square Wave Voltammetry
TELQAS	Target Enrichment Long-Probe Quantitative Amplified Signal Assay
TIMP1	Tissue Inhibitor of Metalloproteinases 1
TNF-α	Tumor Necrosis Factor Alpha
VEGF	Vascular Endothelial Growth Factor
